# Time-lag effects of NEP and NPP to meteorological factors in the source regions of the Yangtze and Yellow Rivers

**DOI:** 10.3389/fpls.2024.1502384

**Published:** 2025-01-10

**Authors:** Hengshuo Zhang, Xizhi Lv, Yongxin Ni, Qiufen Zhang, Jianwei Wang, Li Ma

**Affiliations:** Yellow River Institute of Hydraulic Research, Henan Key Laboratory of Yellow Basin Ecological Protection and Restoration, Zhengzhou, China

**Keywords:** time-lag effects, NEP, NPP, plant functional types, meteorological factors

## Abstract

Vegetation productivity and ecosystem carbon sink capacity are significantly influenced by seasonal weather patterns. The time lags between changes in these patterns and ecosystem (including vegetation) responses is a critical aspect in vegetation-climate and ecosystem-climate interactions. These lags can vary considerably due to the spatial heterogeneity of vegetation and ecosystems. In this study focused on the source regions of the Yangtze and Yellow Rivers (SCRYR), we utilized long-term datasets of Net Primary Productivity (NPP) and model-estimated Net Ecosystem Productivity (NEP) from2015 to 2020, combined with reconstructed 8-day scale climate sequences, to conduct partial correlation regression analysis (isolating the influence of individual meteorological factors on the lag effects). The study found that the length of lag effects varies depending on regional topography, vegetation types, and the sensitivity of their ecological environments to changes in meteorological factors. In the source region of the Yangtze River (SCR), the lag times for NPP and NEP in response to temperature (Tem) are longer, compared to the source region of the Yellow River (SYR), where the lags are generally less than 10 days. The long lag effects of NPP with precipitation (Pre), ranging from 50 to 60 days, were primarily concentrated in the northwestern part of the SCR, while the long lag effects of NEP with precipitation, ranging from 34 to 48 days, covered a broad region in the western part of the study area. NPP exhibits the least sensitivity to changes in solar radiation (SR), with lag times exceeding 54 days in 99.30% of the region. In contrast, NEP showed varying lag effects with respect to SR: short lag effects (ranging from 0 to 15 days) were observed in carbon source areas, while long lag effects (ranging from 55 to 64 days) were evident in carbon sink areas. The sensitivity of vegetation to meteorological changes is highest for SVL, followed by C3A, PW, BDS, and C3 in descending order. This study examined the spatiotemporal impacts of climatic drivers on NPP and NEP from both vegetation and ecosystem perspectives. The findings are crucial for enhancing vegetation productivity and ecosystem carbon sequestration capacity at important water sources in China.

## 1 Introduction

Vegetation and ecosystems are pivotal in carbon sequestration, substantially contributing to the global carbon cycle (Schmitz et al., 2003; de Jong et al., 2013; Wang et al., 2023b; Long et al., 2024). To address global warming, the Chinese government has formulated a series of ecological policies, particularly focusing on the ecological protection and restoration projects in the ecologically vulnerable areas of the Qinghai-Tibet Plateau (Wang et al., 2020; Zhang et al., 2021) and the Sanjiangyuan region (Bian et al., 2017; Wang et al., 2023b), laying a solid foundation for achieving the dual carbon goals (Pei et al., 2009; Li et al., 2022; Zhao et al., 2024). Plant absorb carbon dioxide (CO_2_) from the atmosphere through photosynthesis, leading to the accumulation of organic carbon in biomass, quantified as Net Primary Productivity (NPP) (Wang et al., 2023b; Zhu et al., 2023; Jia et al., 2024). This measure of plant-level productivity is a key component of an ecosystem’s carbon sink function, representing the primary pathway through which carbon is introduced into ecosystems (Yang et al., 2020; Li et al., 2023; Lyu et al., 2023). Beyond NPP, the broader metric of Net Ecosystem Productivity (NEP) encompasses not only the carbon sequestered by plants but also the carbon emissions from the respiration of all biotic components, including soil microorganisms (Ye et al., 2022; Lyu et al., 2023; Zhang et al., 2023a), i.e. NEP provides a comprehensive assessment of the balance between carbon inputs and outputs within an ecosystem, indicating its net role as a carbon sink or source (Ye et al., 2022; Huang et al., 2024).

The efficacy of vegetation and ecosystems in sequestering carbon is modulated by various factors, including species composition (Jobbágy and Jackson, 2000; Pei et al., 2009; Zhao et al., 2024), soil properties (Zhang et al., 2013; Koranda et al., 2023), and climatic conditions (Schmitz et al., 2003; Jones and Driscoll, 2022). Meteorological variables such as temperature (Tem), precipitation (Pre), and solar radiation (SR) are particularly influential (Zhang et al., 2014; Guo et al., 2020; Lyu et al., 2023), as they directly impact physiological processes such as photosynthesis and respiration (Yu, 2020; Liu et al., 2023b). However, the responses of NPP and NEP to these climatic drivers are not immediate, but rather, they often exhibit time-lag effects (Kong et al., 2020; Liu et al., 2021; Huang et al., 2024). These lag effects arise from the complex interactions between biotic and abiotic factors, resulting in a delayed response of carbon sequestration processes to changes in meteorological conditions (Liu et al., 2022; Li et al., 2023; Huang et al., 2024). A comprehensive understanding of these temporal dynamics is essential for accurately forecasting ecosystem responses to climatic variability and for informing strategies aimed at mitigating the effects of climate change (Kong et al., 2020; Huang et al., 2024).

Most previous studies on the time-lag effects of NPP and NEP in response to meteorological factors have typically been conducted at a monthly scale, constrained by data availability and methodological limitations, such as coarse spatial resolution (Liu et al., 2021, 2022; Huang et al., 2024). Early research demonstrated that the relationship between vegetation productivity and climatic variables, such as temperature and precipitation, often exhibited time lags. For instance, Li et al. (2023) observed that in the Qinghai-Tibet Plateau regions, vegetation productivity exhibited a lagged response to climatic variables: up to one month for temperature changes, up to 1.5 months for precipitation changes, and up to two months for changes in solar radiation. Research on the lag effects between NEP and meteorological factors is relatively scarce. This is attributed to the complexity and heterogeneity of the ecosystem processes involved, and the high uncertainty associated with modeling these interactions (Huang et al., 2024). In summary, these studies used of coarse-scale data introduces significant uncertainties, potentially masking finer-scale variations and the intricate interplay between different climatic factors and ecosystem responses (Nicholson and Farrar, 1994; Kong et al., 2020).

The source regions of the Yangtze and Yellow Rivers (SCRYR) play a crucial role in China’s hydrological and ecological systems, serving as the headwaters for two of the country’s most significant rivers (Chen et al., 2020a; Sun et al., 2020; Zhu et al., 2023). These regions are characterized by diverse climatic conditions, ranging from the cold alpine climate of the Tibetan Plateau to the relatively temperate environments at lower elevations (Zhu et al., 2023; Lu et al., 2024). Overall, the Yangtze River source region (SCR) has a higher average elevation, steeper terrain, and colder climatic conditions (Bian et al., 2017; Lu et al., 2024) compared to the Yellow River source region (SYR), which has relatively lower elevations, gentler terrain, and milder climate (Lu et al., 2024; Zhang et al., 2024). These differences lead to variations in climatic conditions, water resource distribution, and vegetation patterns between the two regions (Bian et al., 2017; Kong et al., 2020; Lu et al., 2024). Consequently, they introduce multiple uncertainties in NPP and NEP as well as their lag effects.

Understanding the lag effects of these meteorological factors on NPP and NEP is critical for predicting how these ecosystems might respond to future climate change scenarios (Lyu et al., 2023). In the SCRYR region, where climatic conditions are highly variable and ecosystems are sensitive to environmental changes, examining these lag effects can provide valuable insights into the resilience and adaptability of the region’s ecosystems (Kong et al., 2020; Liu et al., 2022; Huang et al., 2024). Moreover, different plant functional types (PFTs) within these ecosystems may exhibit distinct responses to meteorological factors, further complicating the overall carbon dynamics (Wang and Ni, 2005; Rao et al., 2024). The study aims to 1) investigate the finer-scale lag effects of temperature, precipitation, and solar radiation on NPP and NEP at a daily scale in the SCRYR region; 2) By analyzing these lag effects from both vegetation and ecosystem perspectives, along with their spatiotemporal variations and relationships with different PFTs, we seek to enhance the understanding of the mechanisms underlying carbon sequestration and release (mainly time lag variations) in this ecologically significant area. Our research provided a scientific basis for understanding the response of the ecosystem in the source regions of the Yangtze and Yellow Rivers to climate change, revealing adaptive strategies for carbon transformation and changes in productivity. This work offers theoretical support and practical guidance for further optimizing ecosystem management measures and developing regional climate change adaptation plans.

## 2 Materials and methods

### 2.1 Study area

**Figure 1:**
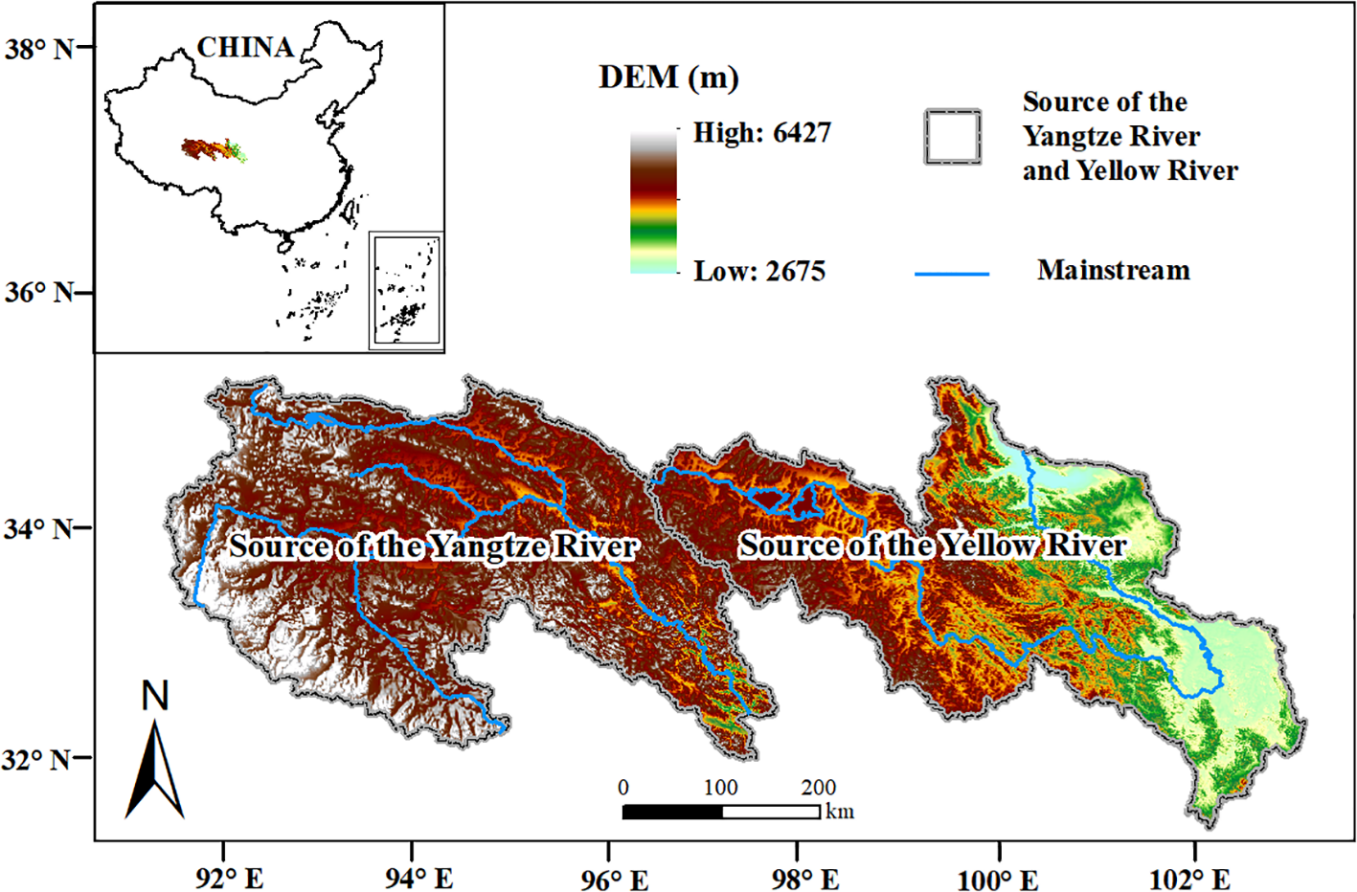
Location of the study area.

The SCRYR are located in the northeastern section of the Qinghai-Tibet Plateau in China (**Figure 1**), spanning from 89°49′ to 103°29′E and 31°18′ to 36°56′ N. The elevation of this region ranging from 2675 to 6427 m and covers a total area of 264389 km^2^. The SCRYR share borders with Gansu Province to the northeast and the Tibet Autonomous Region to the west and south. To the southeast, they connect to Sichuan Province, and within the central area, they encompass parts of Qinghai Province, including the Yushu Tibetan Autonomous Prefecture and the Golog Tibetan Autonomous Prefecture. The SCRYR is the source of two important rivers in Asia, i.e., the Yangtze River and Yellow River, and is also a vital water source and significant natural resource conservation area in China (Wang et al., 2021b, 2023; Zhu et al., 2023).

The SCRYR has a continental plateau climate, with annual precipitation ranging from 200 to 550 mm (increasing gradually from west to east) and an average annual temperature from -4°C to 5°C (due to the influence of elevation, temperatures in higher elevation areas are significantly lower) (Sun et al., 2020; Wang et al., 2021b, 2023). Due to its unique geographical location and relatively thin atmosphere, the SCRYR experiences higher solar radiation intensity, ample sunlight during the summer and significant seasonal variability (Liu et al., 2012; Yu et al., 2022). As a result, the vegetation in these high- elevation regions is highly sensitive to variations in temperature (Tem), precipitation (Pre), and solar radiation (SR) (Fan and Bai, 2021; Xu and Wu, 2023).

**Figure 2:**
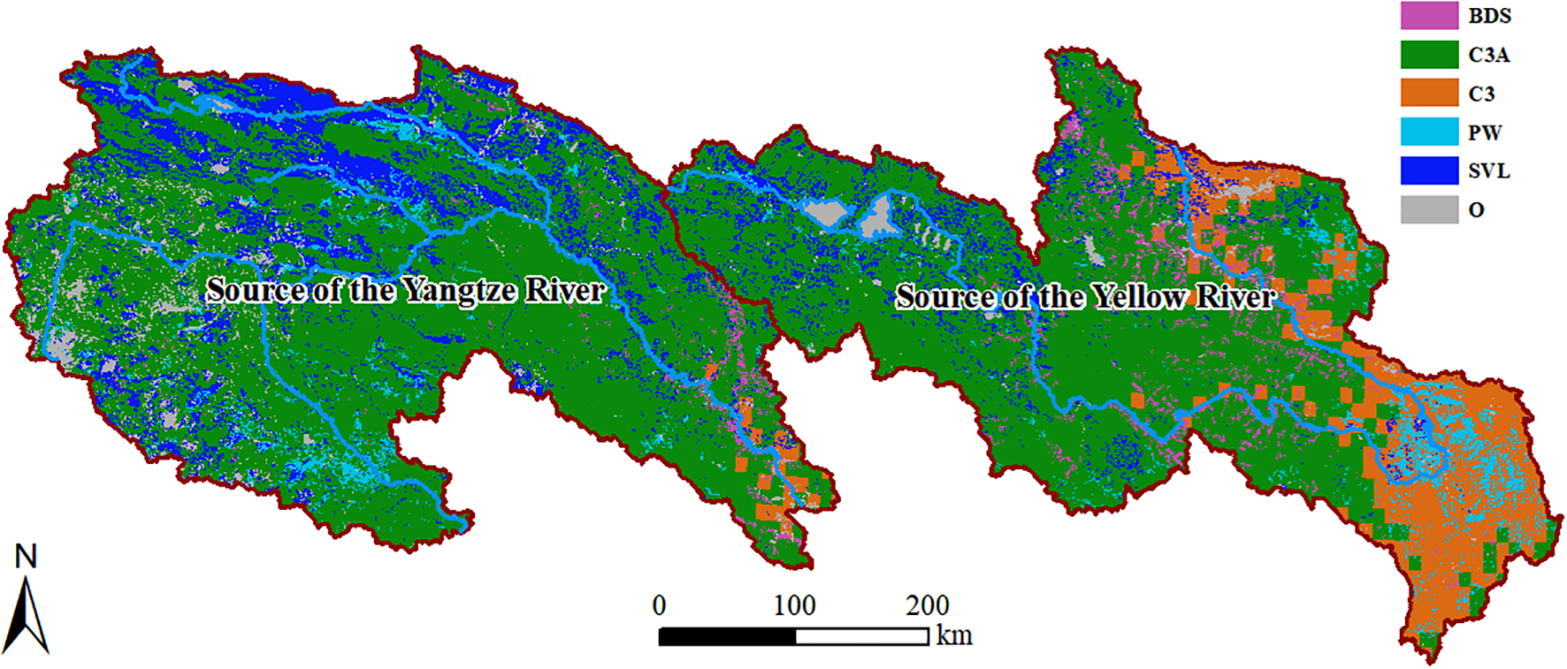
Distribution of PFTs in the SCRYR.

Generally, the plant functional types (PFTs) in the SCRYR (**Table 1**) primarily include Broadleaf deciduous shrub, boreal (BDS, accounting for 2.65% of the total area), C3 grass, arctic (C3A, accounting for 67.40% of the total area), C3 grass (C3, accounting for 8.63% of the total area), and Permanent wetlands (PW, accounting for 3.55% of the total area), with additional presence of sparsely vegetated lands (SVL, accounting for 13.39% of the total area). Other types (O, accounting for 4.37% of the total area), such as crops, water bodies, and urban construction land, are irregularly distributed throughout the study area. The primary ecosystem type in both the SCRYR is C3A (**Figure 2**). The distinction lies in the distribution of SVL, which is more prevalent in the source of Yangtze River region (in the northern part), while C3 is more commonly found in the source of Yellow River region (in the southeastern part). The distribution of ecosystem types is influenced by climate, topography, and human activities (Fan and Bai, 2021; Xu and Wu, 2023).

**Table 1:** Area of PFTs in the SCRYR (source of the Yangtze River and Yellow River).

Number	Name	Abbreviation	Area/km^2^
1	Broadleaf deciduous shrub, boreal	BDS	7017.75
2	C3 grass,arctic	C3A	178201.00
3	C3 grass	C3	22823.50
4	Permanent wetlands	PW	9389.00
5	sparsely vegetated lands	SVL	35392.00
6	Other types	O	11565.75

### 2.2 Data sources

#### 2.2.1 NPP data sources

The NPP data used in this study was obtained from the 2015 to 2020 MOD17A2H 8-days composite datasets with 500m spatial resolution from the US NASA EOS/MODIS (https://search.earthdata.nasa.gov/). In addition, the dataset also eliminates cloud and snow pollution interference, and the quality control files of the dataset clean up the lower quality data, increasing the reliability of NPP data (Gong, 2021; 2022). The original NPP data (MOD17A2H) was in the Hierarchical Data Format (HDF). To extract the data within the study area, the MODIS Reprojection Tool was utilized to convert the set of HDF data into TIFF format and project them using the SCRYR boundary vector file as a mask. The MOD17A2H product outliers were removed, and the effective values were multiplied by the product scaling factor of 0.0001, by the MOD17A2H product proportion factor in the MOD17 User’s Guide, to obtain the NPP data in units of g C·m^−2^·8d^−1^.

#### 2.2.2 Meteorological data sources

Meteorological data (including Tem, Pre) were obtained from the National Tibetan Plateau Data Center (https://data.tpdc.ac.cn/). Tem data (°C) were derived from the TRIMS LST-TP dataset (Zhang et al., 2023b), and the spatial resolution is 1 km, with a daily temporal resolution. Pre data (mm) were derived from the CHM_PRE dataset (Han et al., 2023), and the spatial resolution is 0.1°, with a daily temporal resolution. SR data (W·m^-2^) were obtained from the fifth generation of ECMWF atmospheric reanalysis data set for the global climate (ERA5, https://cds.climate.copernicus.eu/), and the spatial resolution is 0.25°, with a hourly temporal resolution. Through projection transformation, resampling, cropping, and arithmetic calculations, Tem and Pre grid data for the SCRYR from 2015 to 2020 were derived at a spatial resolution of 500 m. All grid data in this study has been unified to the geographic coordinate system (Lohmar, 1988).

#### 2.2.3 DEM data sources

Elevation data were obtained from the ASTER Global Digital Elevation Model V002 (https://search.earthdata.nasa.gov/). The original spatial resolution is 250m (dated 2020), through projection transformation, resampling, and mask extraction, DEM grid data for the SCRYR were derived at a spatial resolution of 500 m.

#### 2.2.4 PFTs data sources

PFTs data were obtained from the National Tibetan Plateau Data Center (https://data.tpdc.ac.cn/) (Ran and Li, 2019). The original spatial resolution is 1km, through projection transformation, resampling, and mask extraction, PFTs grid data for the SCRYR were derived at a spatial resolution of 500 m.

### 2.3 Methods

#### 2.3.1 Evaluation of vegetation carbon sink capacity in SCRYR based on NPP

NEP plays a crucial role in the material and energy flows of ecosystems. It represents the capacity of plant communities to produce carbon under natural environmental conditions and serves as a fundamental indicator for evaluating the coordination of ecosystem structure and function, as well as the biosphere’s carrying capacity (Yu, 2020; Lyu et al., 2023). NEP is calculated as the difference between NPP and ecosystem respiration (*R_h_*).


(1) $$NEP=NPP-R_{h}$$

We employed the methodology developed by Pei et al. (2009), which integrates Pre, Tem, and carbon emissions to establish a regression equation for estimating regional *R_h_*. This approach has demonstrated efficacy in evaluating vegetation NEP within the region and has been successfully applied and validated in the ecosystems of the Qinghai-Tibet Plateau, China (Jiang et al., 2022; Ye et al., 2022).


(2) $$R_{h}=0.22\left({\text{e}}^{0.0912Tem}+\ln(0.3145Pre+1\right))\times 30\times 46.5\%$$

Given that the unit of NPP is g C·m^−2^·8d^−1^, the units for NEP and R_h_ are also g C·m^−2^·8d^−1^. Pre and Tem represent the total Pre (mm) and average Tem (°C) over an 8-day period, respectively.

#### 2.3.2 Partial correlation analysis

This study analyzed the response of NEP to meteorological factors (Tem, Pre, and SR) by using the partial correlation method (Lyu et al., 2023; Zhang et al., 2023a). The computation of partial correlation coefficient (PCC), while controlling for two or more variables, generally involves employing multiple linear regression to mitigate the influence of the control variables. In this study, the `*partialcorr*` function in MATLAB R2023b was utilized to perform these calculations. This approach enabled us to derive partial correlation coefficients, which were subsequently used to assess the impact of Pre, Tem, or Solar on NPP (or NEP) (Lyu et al., 2023). The statistical significances of the regression and partial correlation coefficients were examined using the T test, and the p-values less than 0.05 were considered significant (Zheng et al., 2020).

#### 2.3.3 Lag effect analysis

**Figure 3:**
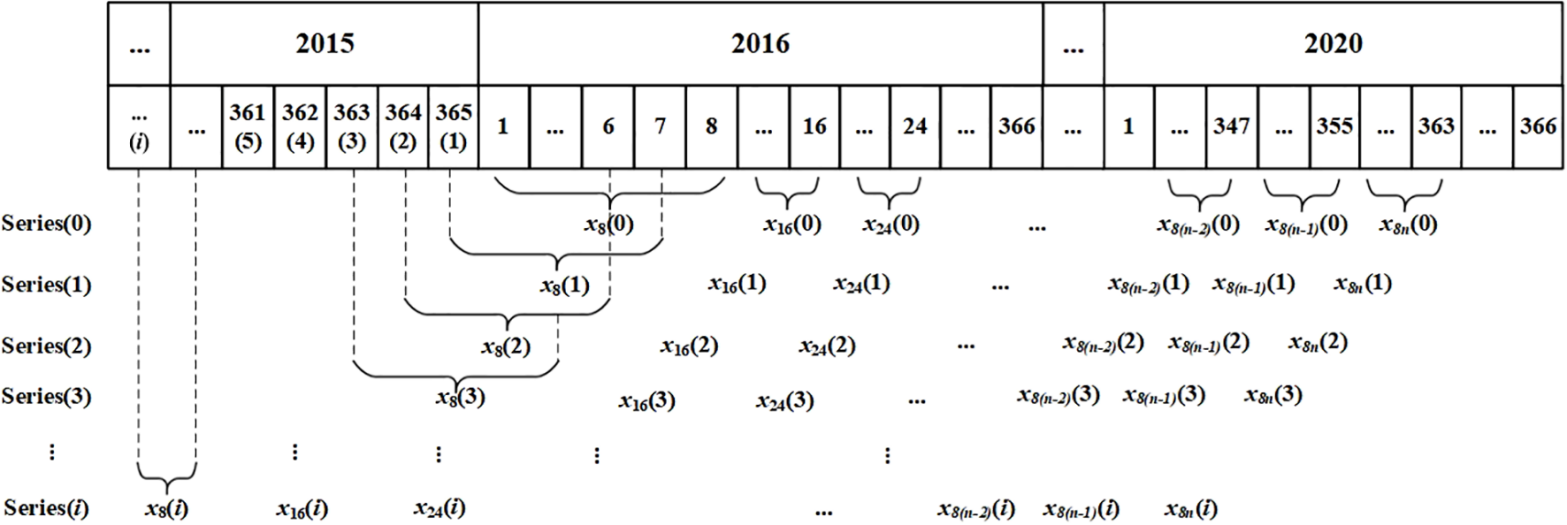
Sketch illustrating how 8-day scale meteorological data were reconstructed using a moving average calculated over a sliding time window.

We acquired NPP and calculated NEP datasets at an 8-day temporal resolution. To investigate the time-lag relationships between 8-day scale NPP (or NEP) and Tem, Pre, and SR, we employed a novel and straightforward approach. This method involved reconstructing new 8-day scale temperature, precipitation, and solar radiation series with a time lag of *i* days by advancing the start date of the original time series by *i* days. Previous studies have indicated that the time lag between Tem, Pre, or SR and NPP in the north of China were no great than three months (Liu et al., 2021, 2022; Huang et al., 2024). Considering this and the preliminary peak value analysis of NPP, NEP, TEM, Pre, and SR in our study, we examined time lags ranging from 0 to 120 days [i.e., (*i* = 0, 1, 2, 3, …, 120)]. Subsequently, we reconstructed the 8-day scale Tem, Pre, and SR based on the restructured daily scale sequences (**Figure 3**). Specifically, the reconstructed 8-day scale Pre was the sum of daily Pre, the reconstructed 8-day scale Tem was the average of daily Tem, and the reconstructed 8-day scale SR was the average of daily SR. Subsequently, we used MATLAB R2023b to repeatedly perform the partial correlation analysis described in Section 2.3.2 to determine the relationships between 8-day scale NPP (or NEP) and 8-day scale climatic factors. For Tem and Pre, the lag days (*i*) with the highest PCC were identified as the time lag days required for NPP (or NEP) to respond to the respective factor. For SR, the lag days (*i*) with the lowest PCC were identified as the time lag days required for NPP (or NEP) to respond to the respective factor.

#### 2.3.4 Pearson correlation analysis

Using MATLAB R2023b, we generated new gridded data for the lag days (*i*) of NPP (or NEP) and meteorological factors (Tem, Pre, and SR). We then extracted the lag days (*i*) for each grid cell and performed Pearson correlation analysis with the latitude, longitude, and elevation of the respective grid cell. This analysis aimed to investigate the spatial distribution differences of the lag effects of NPP and NEP in the SCRYR.

## 3 Results

### 3.1 Spatiotemporal distribution characteristics of NPP and NEP

**Figure 4:**
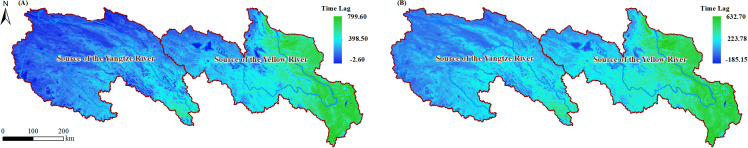
The annual average values of NPP **(A)** and NEP **(B)** from 2015 to 2020 in the SCRYR.

**Figure 5:**
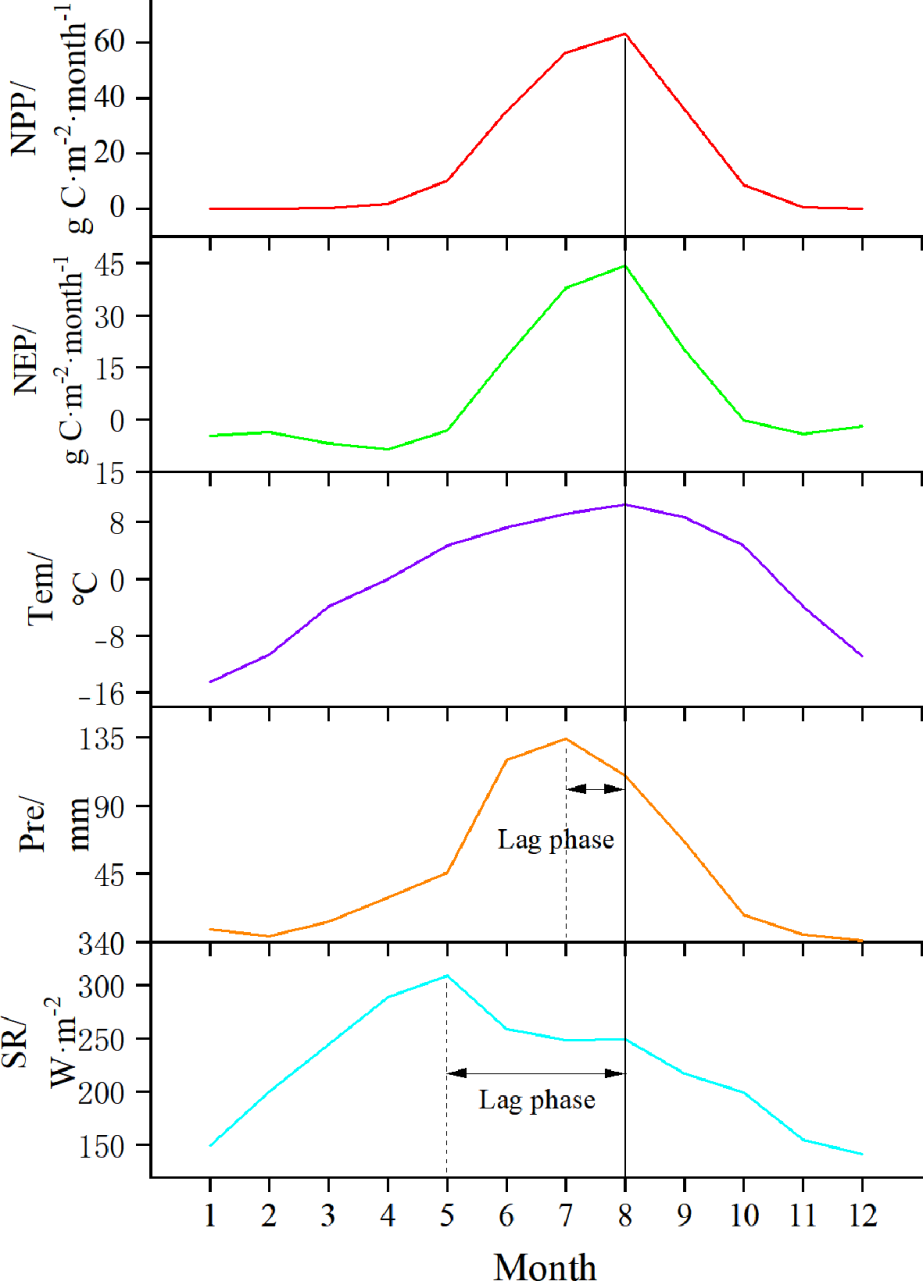
The monthly scale variation of NPP, NEP, Tem, Pre, and SR from 2015 to 2020 in the SCRYR.

To analyze the spatial distribution characteristics of NPP and NEP, we aggregated the 8-day scale NPP and NEP datasets from 2015 to 2020 into multi-year averages and generated new gridded data (**Figure 4**). To analyze the temporal variation trends of NPP and NEP, we organized the 8-day NPP and NEP datasets from 2015 to 2020 into monthly averages and compared them with monthly scale meteorological factor values (**Figure 5**).

As shown in **Figure 4A**, during the period from 2015 to 2020, the annual mean NPP in the SCRYR ranged from -2.60 to 799.60 g C·m⁻²·a⁻¹, exhibiting significant variation with longitude, increasing from west to east. Specifically, the annual mean NPP in the SCR was approximately 134.10 g C·m⁻²·a⁻¹, while in the SYR, it was approximately 302.18 g C·m⁻²·a⁻¹. The annual mean NEP in the SCRYR ranged from -185.15 to 632.70 g C·m⁻²·a⁻¹ (**Figure 4B**), which exhibited a spatial variation trend similar to that of NPP. From northwest to southeast, there is a gradual transition from carbon source regions (NEP <0) to carbon sink regions (NEP >0). The annual mean NEP in the SCR was approximately 20.25 g C·m⁻²·a⁻¹, while in the SYR, it was approximately 164.11 g C·m⁻²·a⁻¹.

From a temporal perspective, the variation trends of NPP and NEP in the study area were similar (**Figure 5**). Both increased sharply from May to July, reached their peak in August (63.12 g C·m⁻²·month⁻¹and 44.20 g C·m⁻²·month⁻¹, respectively), and then declined rapidly. Here we also focused on the monthly scale variation trends of meteorological factors (i.e. Tem, Pre, and SR). Tem reached its peak in August (10.48°C), coinciding with the peak values of NPP and NEP. Pre peaked in July (134.13mm), while SR peaked in May (308.94 W·m^-2^). Consequently, the peaks of NPP and NEP lag behind Pre by approximately one month and SR by about three months. Additionally, it is noteworthy that while Pre and NPP/NEP peak simultaneously on a monthly scale, this only may indicate a more pronounced lag effect on a daily scale.

**Figure 6:**
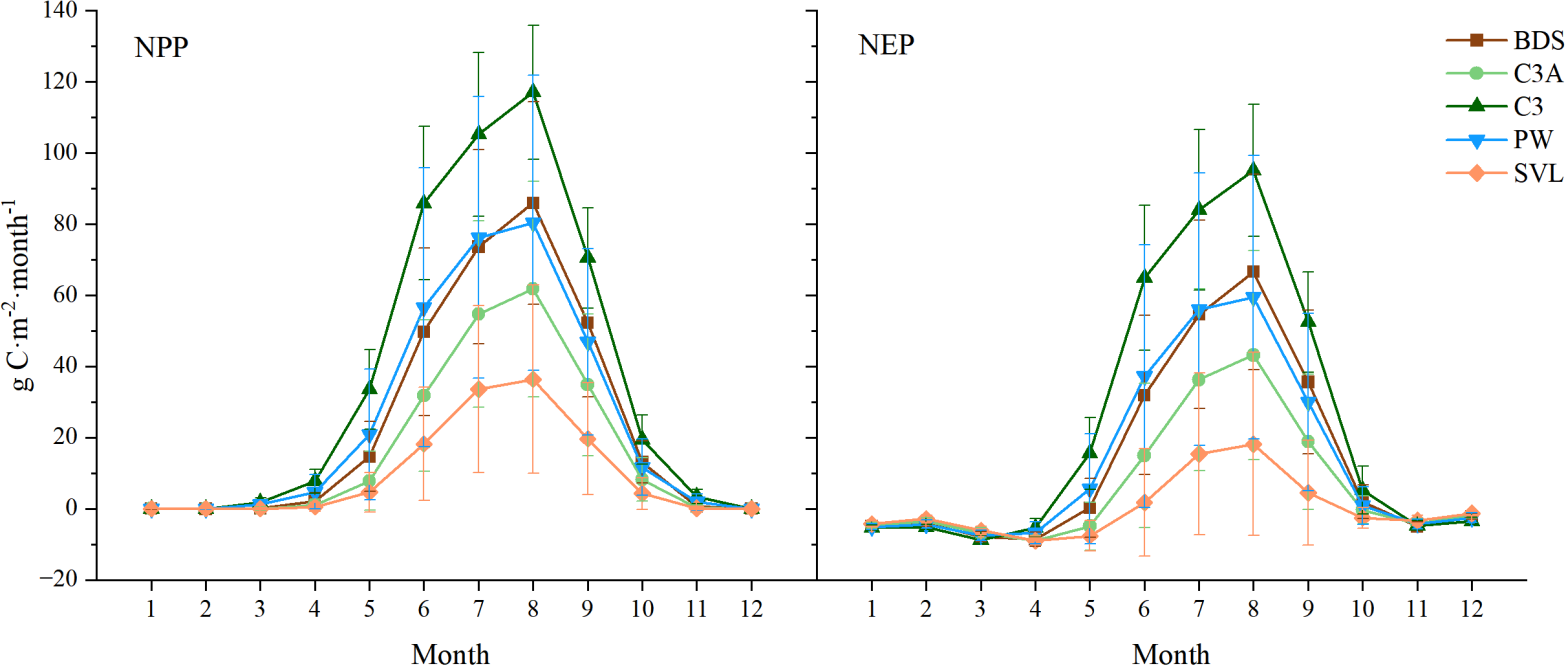
Monthly variation of NPP and NEP of different PFTs in the SCRYR. error bars represent SD (standard deviation, 95% confidence intervals).

We further analyzed the monthly variation of NPP and NEP values across different PFTs (**Figure 6**) and found that the variation trends for NPP and NEP under different PFTs were almost identical to the overall monthly variation trends in the study area (**Figure 5**). However, there were notable differences in the amplitude of fluctuations. Specifically, the NPP peak in August followed the order of C3 (117.00± 18.80 g C·m⁻²·month⁻¹) > BDS (85.88± 28.39 g C·m⁻²·month⁻¹) > PW (80.32± 41.48 g C·m⁻²·month⁻¹) > C3A (61.73± 30.24 g C·m⁻²·month⁻¹) > SVL (36.39± 26.46 g C·m⁻²·month⁻¹), and the NEP peak followed the same order: C3 (95.02± 18.56 g C·m⁻²·month⁻¹) > BDS (66.56± 27.46 g C·m⁻²·month⁻¹) > PW (59.39± 39.84 g C·m⁻²·month⁻¹) > C3A (43.17± 29.39 g C·m⁻²·month⁻¹) > SVL (18.13± 25.74 g C·m⁻²·month⁻¹).

### 3.2 Lagged responses of vegetation to meteorological factors

**Figure 7:**
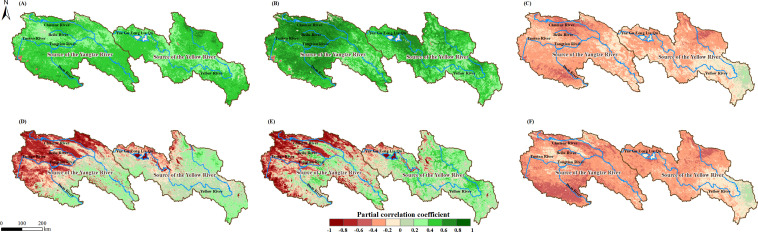
Spatial distributions of PCC between NPP and Tem **(A)**, Pre **(B)**, and SR **(C)** in the SCRYR; spatial distributions of PCC between NEP and Tem **(D)**, Pre**(E)**, and SR **(F)** in the SCRYR; scenario without time lag.

Meteorological factors from the normal time series (scenario without time lag) were used as independent and control variables, with NPP and NEP as dependent variables for partial correlation analysis. The spatial distribution of the PCC is shown in **Figure 7**. NPP showed a positive feedback effect to increases in Tem (**Figure 7A**) and Pre (**Figure 7B**) across the entire study area, with PCC ranges of -0.23 to 0.78 and -0.39 to 0.94, respectively. Conversely, NPP exhibited a negative feedback effect to increases in SR across most of the study area, with a PCC range of -0.63 to 0.38. In contrast, NEP’s response to Tem and Pre exhibited significant regional differences within the study area, transitioning from negative feedback effects in the northwest to positive feedback effects in the southeast, with PCC ranges of -0.94 to 0.60 and -0.95 to 0.76, respectively. Similar to NPP, NEP also showed a negative feedback effect to increases in SR across most of the study area, with a PCC range of -0.69 to 0.38.

**Figure 8:**
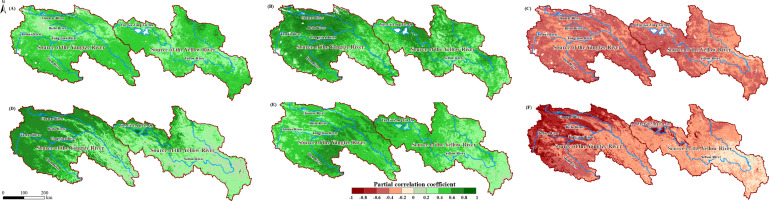
Maximum (or minimum) PCC between the NPP and Tem **(A)**, Pre **(B)** and SR **(C)** in the SCRYR; maximum (or minimum) PCC between the NEP and Tem **(D)**, Pre **(E)** and SR **(F)** in the SCRYR; scenario with time lag.

We performed partial correlation analysis between the NPP and NEP data and the reconstructed time series of meteorological factors. The spatial distribution of the partial correlation coefficients is shown in **Figure 8**. Within the study area, the responses of NPP and NEP to meteorological factors were corrected. The PCC range for NPP and Tem was corrected to -0.13 to 0.78, for NPP and Pre to 0.02 to 0.94, and for NPP and SR to -0.74 to 0.09. For NEP, the PCC range with Tem was corrected to -0.11 to 0.85, with Pre to 0.05 to 0.86, and with SR to -0.17 to -0.89.

To analyze the differences in PCC between NPP (or NEP) and meteorological factors across different PFTs, we conducted further analysis under both scenarios: without time lag and with time lag (**Table 2**). The results showed that under the scenario without time lag, the PCC between NPP (or NEP) and meteorological factors was not sensitive to changes in PFTs. However, under the scenario with time lag, the PCC between NEP and Tem was most sensitive to PFT changes. The mean PCC values were ranked as follows: SVL (0.63) > C3A (0.46) > PW (0.40) > BDS (0.33) > C3 (0.26). The next most sensitive was the PCC between NEP and SR: SVL (-0.58) < C3A (-0.39) < PW (-0.36) < BDS (-0.30) < C3 (-0.21).

**Table 2:** PCC between NPP (or NEP) and Tem, Pre, and SR in the SCRYR.

		Without time lag						With time lag					
		NPP			NEP			NPP			NEP		
		Tem	Pre	SR	Tem	Pre	SR	Tem	Pre	SR	Tem	Pre	SR
BDS	Min	0.08	-0.16	-0.56	-0.91	-0.94	-0.64	0.08	0.05	-0.68	-0.08	0.09	-0.86
	Max	0.73	0.87	0.25	0.58	0.74	0.20	0.73	0.87	-0.09	0.85	0.76	0.07
	Mean	0.44	0.48	-0.22	0.07	0.16	-0.27	0.44	0.48	-0.40	0.33	0.44	-0.30
C3A	Min	-0.13	-0.19	-0.62	-0.93	-0.95	-0.69	-0.13	0.02	-0.74	-0.11	0.06	-0.88
	Max	0.77	0.93	0.22	0.60	0.76	0.19	0.77	0.93	-0.06	0.85	0.85	0.09
	Mean	0.45	0.54	-0.24	-0.08	-0.02	-0.31	0.45	0.54	-0.44	0.46	0.49	-0.40
C3	Min	-0.03	-0.14	-0.59	-0.93	-0.88	-0.61	-0.01	0.02	-0.68	-0.09	0.05	-0.83
	Max	0.78	0.84	0.38	0.60	0.71	0.38	0.78	0.84	0.09	0.81	0.74	0.17
	Mean	0.46	0.46	-0.12	0.17	0.26	-0.14	0.46	0.46	-0.37	0.26	0.34	-0.21
PW	Min	-0.14	-0.10	-0.59	-0.94	-0.93	-0.69	-0.02	0.09	-0.70	-0.06	0.06	-0.89
	Max	0.74	0.89	0.36	0.52	0.72	0.36	0.74	0.89	-0.01	0.84	0.85	0.15
	Mean	0.47	0.53	-0.20	-0.01	0.04	-0.22	0.47	0.53	-0.41	0.40	0.44	-0.36
SVL	Min	-0.16	-0.19	-0.63	-0.92	-0.94	-0.68	-0.13	0.09	-0.70	-0.03	0.09	-0.89
	Max	0.77	0.94	0.26	0.54	0.75	0.27	0.77	0.94	0.01	0.85	0.86	0.12
	Mean	0.45	0.62	-0.29	-0.40	-0.34	-0.30	0.45	0.62	-0.41	0.63	0.49	-0.58

### 3.3 Distribution of vegetation time lags

**Figure 9:**
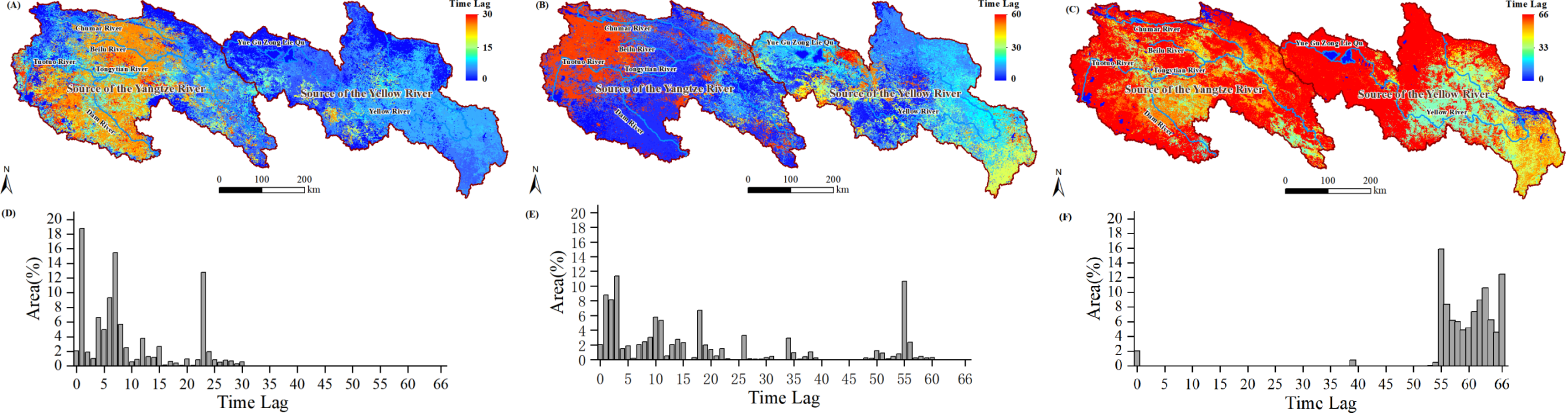
Time lags at which there was maximum PCC between the NPP and Tem **(A)** and Pre **(B)**, and minimum PCC between the NPP and SR **(C)**; histogram of time lags between the NPP and Tem **(D)**, Pre **(E)**, and SR **(F)**.

**Figure 10:**
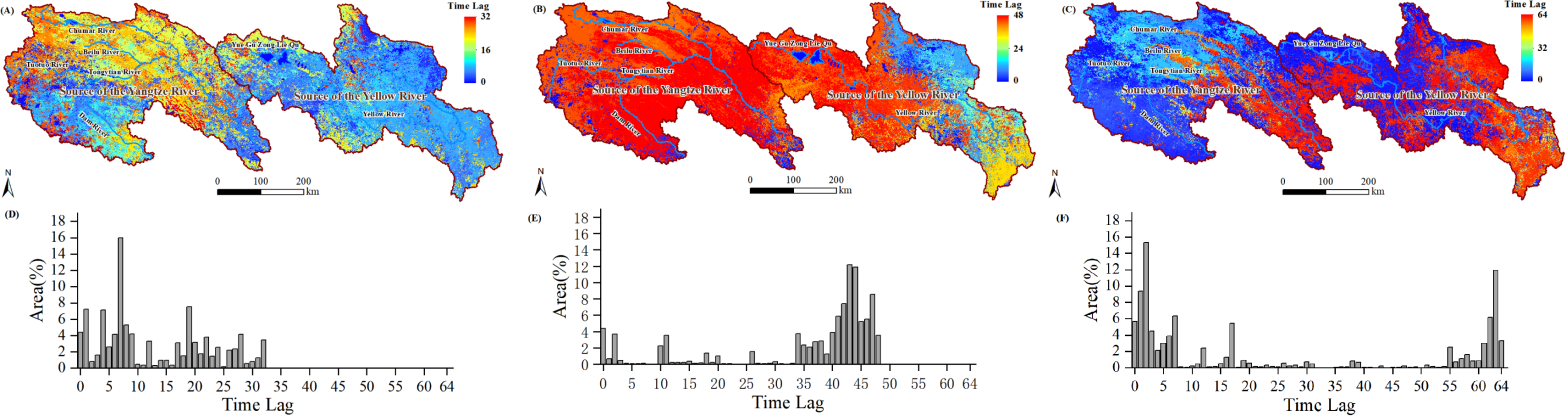
Time lags at which there was maximum (minimum) PCC between the NEP and Tem **(A)**, Pre **(B)**, and minimum PCC between the NEP and SR **(C)**; histogram of time lags between the NEP and Tem **(D)**, Pre **(E)**, and SR **(F)**.

To further investigate the distribution characteristics of the lag effects of NPP and NEP within the study area, we extracted the lag days corresponding to the maximum (or minimum) PCC values and re-mapped them as grid images (**Figures 9A–C**; **Figures 10A–C**). The corresponding histograms are shown in **Figures 9D–F** (also in **Figures 10D–F**).

The lag relationship between NPP and Tem showed a spatial pattern of higher lag days in the west and lower lag days in the east (**Figure 9A**, i.e., most areas in SCR have longer lag days compared to the SYR region). The histogram data revealed that in the SCR region, lag days predominantly range from 20 to 30 days, whereas in the SYR region, lag days were primarily between 0 and 10 days (**Figure 9D**). The lag relationship between NPP and Pre indicated that areas with lag days ranging from 0 to 30 account for 76% of the study area (**Figure 9E**), while areas with lag days ranging from 50 to 60 account for 17.57% of the study area and were primarily concentrated in the northwest part of the SCR (**Figure 9B**). The lag days between NPP and SR were primarily concentrated in the 54 to 66 range. (**Figure 9F**, 99.30% of the study area).

The lag relationship between NEP and Tem exhibited a spatial distribution similar to that of the lag relationship between NPP and Tem (**Figure 10A**). In the SCR region, the lag days predominantly range from 12 to 32 (**Figure 10D**), whereas in the SYR region, the lag days mainly range from 0 to 10. The lag relationship between NEP and Pre indicated that areas with lag days greater than 34 account for 85.72% of the study area (**Figure 10B, E**), while only 14.23% of the area had lag days less than 30 (primarily concentrated in the northeastern part of the SYR region). The lag relationship between NEP and SR in the SCRYR region exhibited a bimodal distribution (**Figure 10C, F**), with lag days predominantly concentrated in the 0 to 15 days (northwest region) and 55 to 64 intervals (southeast region).

As previously analyzed, there were spatial differences in the lag effects between NPP (or NEP) and meteorological factors. Therefore, we extracted the lag days for each grid cell along with their corresponding latitude, longitude, and elevation values, and conducted Pearson correlation analysis (**Table 3**). The results showed that the lag effects of NPP with Tem were highly significantly negatively correlated with longitude (*r*= -0.45, P< 0.01). For NEP, the lag effects with Tem and Pre factors were highly significantly negatively correlated with longitude (the *r* values were -0.39 and -0.37, respectively). In contrast, the lag effects of NPP (or NEP) with the three meteorological factors showed little correlation with latitude (only the lag effect between NEP and SR shows a significant correlation, and the *r* values was 0.40). The lag effects of NPP with Tem were highly significantly positively correlated with elevation (*r*= 0.30, P< 0.01), and the lag effects of NEP with the Tem and Pre factors were also highly significantly positively correlated with elevation (the *r* values were 0.38 and 0.41, respectively). While the lag effects of NEP with SR were negatively correlated with elevation (*r*= 0.-36, P< 0.01).

**Table 3:** Pearson correlation between lag effects and latitude, longitude, elevation.

	Longitude	Latitude	Elevation
Lag_ NPP& Tem	-0.45	0.07	0.30
Lag_ NPP& Pre	-0.10	-0.13	0.04
Lag_ NPP& SR	-0.27	0.08	0.24
Lag_ NEP& Tem	-0.39	-0.26	0.38
Lag_ NEP& Pre	-0.37	-0.10	0.41
Lag_ NEP& SR	0.24	0.40	-0.36

P< 0.01; Lag_ NPP& Tem represented the lag days of NPP and Tem; Lag_ NPP& Pre represented the lag days of NPP and Pre; Lag_ NPP& SR represented the lag days of NPP and SR; Lag_ NEP& Tem represented the lag days of NEP and Tem; Lag_ NEP& Pre represented the lag days of NEP and Pre; Lag_ NEP& SR represented the lag days of NEP and SR; the same below.

### 3.4 Differences in lag effects among various PFTs

As shown in Section 3.1, there were significant differences in the monthly scale NPP and NEP across different PFTs within the SCRYR region. Therefore, to exam the time-lag effects and their variability across various PFTs, we conducted further analysis (**Supplementary Figure S1**–**S6**).

The average lag days of NPP with Tem across different PFTs were ranked as follows (**Table 4**): PW (11.25)> SVL (10.29)> C3A (9.83)> BDS (6.66)> C3 (5.65). For BDS, the area proportion with lag days in the 0 to 8 range was 85.12% (**Supplementary Figure S1F**), whereas for C3, the area proportion within this lag range was 97.65% (**Supplementary Figure S1H**). The average lag days for BDS and C3 are significantly lower than those for C3A, PW, and SVL, which is closely related to the spatial distribution of PFTs. Over 20% of the area for C3A, PW, and SVL is located in the western part of the SCR, with lag days ranging from 22 to 25 (**Supplementary Figures S1G, I, J**). The average lag days of NPP with Pre across different PFTs were ranked as follows: C3 (21.90)> SVL (20.34)> C3A (18.46)> PW (17.39)> BDS (15.07). Influenced by the spatial distribution of PFTs, C3A and SVL also exhibit a bimodal lag distribution, with lag days primarily concentrated in the 0 to 15 and 55 to 56 ranges (**Supplementary Figure S2B, E, G, J**; C3A and SVL with larger lag days were primarily distributed in the northwest part of the SCR). Meanwhile, the lag days for C3A, PW, and BDS were mainly within the 0 to 35 range (**Supplementary Figure S2F, H, I**; the area proportions were 79.37%, 89.27%, and 91.46%, respectively). The average lag days of NPP with SR across different PFTs were ranked as follows: C3 (15.19)> BDS (12.85)> PW (10.23)> C3A (9.97)> SVL (8.88). Although the lag range in SCRYR spanned from 0 to 50, 99% of the area has lag days within 30 days. For different PFTs, most lag days fall within the 0 to 17 range (**Supplementary Figure S3**). The area proportions within this lag range were 79.97% for BDS, 87.70% for C3A, 65.81% for C3, 82.90% for PW, and 88.20% for SVL.

**Table 4:** Average lag days of NPP (NEP) with meteorological factors across different PFTs.

	BDS	C3A	C3	PW	SVL
Lag_ NPP& Tem	6.66	9.83	5.65	11.25	10.29
Lag_ NPP& Pre	15.07	18.46	21.90	17.39	20.34
Lag_ NPP& SR	58.70	60.80	57.60	61.93	58.86
Lag_ NEP& Tem	9.55	13.70	7.50	12.17	17.19
Lag_ NEP& Pre	31.12	39.63	25.04	32.63	39.43
Lag_ NEP& SR	32.05	25.03	43.47	31.42	14.12

The average lag days of NEP with Tem across different PFTs were ranked as follows: SVL (17.19)> C3A (13.70)> PW (12.17)> BDS (9.55)> C3 (7.50). The lag days of BDS, C3, and PW exhibited a unimodal distribution ((**Supplementary Figure S4F, H, I**)), with the highest proportion of area having a lag of 7 days (accounting for 31.25%, 48.13%, and 27.96% respectively). The proportions of area with lag days in the 0 to 9 range are 77.85% for BDS, 89.63% for C3, and 58.68% for PW. However, the lag ranges for C3A and SVL are relatively evenly distributed within 0 to 32 days (**Supplementary Figures S4G, J**), with the maximum area proportion corresponding to any specific lag day not exceeding 20%. Additionally, for C3A and SVL, which are widely distributed in SCRYR, the lag days are greater in the SCR region compared to the SYR region (**Supplementary Figures S4B, E**). The average lag days of NEP with Pre across different PFTs were ranked as follows: C3A (39.63)> SVL (39.43)> PW (32.63)> BDS (31.12)> C3 (25.04). The lag days of NEP with Pre were distributed within the 34 to 48 range (**Supplementary Figure S5**). The area proportions within this range were 61.99% for BDS, 85.72% for C3A, 46.26% for C3, 68.09% for PW, and 88.78% for SVL. The average lag days between NPP and SR across different PFTs were all around 60 days.

## 4 Discussion

### 4.1 Spatiotemporal dynamics of NPP and NEP

NPP primarily focuses on plant-level productivity and carbon exchange (Jiang et al., 2020; Lyu et al., 2023; Jia et al., 2024), while NEP considers carbon exchange at the entire ecosystem level, including respiration from all biological components and other related biochemical processes (Yu, 2020; Lyu et al., 2023; Zhang et al., 2023a). Therefore, we analyzed the carbon sink capacity of the SCRYR region from both the plant and ecosystem levels.

NPP as the key indicator of plant growth and organic matter accumulation (Zhu et al., 2023; Jia et al., 2024), reflects how much energy the ecosystem can provide to other parts of the food chain, such as animals and microorganisms (Yang et al., 2020; Wang et al., 2023b). The reasons for the significant spatial variation in the annual average NPP in the SCRYR region are multifaceted. NPP in SCRYR is increasing spatially from northwest to southeast, with a mean NPP value range of -2.60 to 799.60 g C·m^−2^·a^−1^ during 2015 to 2020 (**Figure 4A**). This variation aligned with the spatial patterns of precipitation and temperature changes, yet contrasted with the variation in SR (**Supplementary Figure S7**), in agreement with the findings of Zhang et al. (2014); Jiang et al. (2020), and Liu et al. (2023a). In alpine grassland regions, temperature directly enhances photosynthesis (leading to increased vegetation productivity), while increased precipitation provides more available water for vegetation (Guo et al., 2020). In contrast, high radiation intensity often accompanies evapotranspiration from vegetation and soil, thereby reducing the available water for vegetation (Long et al., 2024). Additionally, the NPP of different PFTs exhibited significant variability on a monthly scale (**Figure 6**), and these changes in NPP with latitude, longitude, and elevation exhibited co-variability with vegetation distribution (Zhang et al., 2014, 2021).

This study estimated NEP using a model of the relationship between NPP and NEP that has been widely utilized in previous research (Yu, 2020; Lyu et al., 2023). The variation patterns of NEP, whether in terms of spatial distribution (**Figure 4**), temporal dynamics (**Figure 5**), or changes across different PFTs (**Figure 6**), showed a high degree of similarity with those of NPP. This indicates that the organic matter accumulation capacity at the plant level in the SCRYR region largely determines its carbon sink capacity at the ecosystem level. The high degree of synchronicity between NPP and NEP across spatial and temporal scales indirectly confirms that most of the study area comprises mature and stable ecosystems with minimal human interference (Liu et al., 2024). This synchronicity reflects the tight coupling among ecosystem components and their coordinated response to external changes (Schmitz et al., 2003; Jones and Driscoll, 2022).

### 4.2 Indications of time-lag effects

The productivity and carbon sink capacity within the study area reached its maximum in August (**Figure 5**), both at the vegetation level (as indicated by NPP) and at the ecosystem level (as indicated by NEP). This phenomenon is closely linked to the optimal climatic conditions present within the study area (Wang et al., 2020), the phenological stages of vegetation (Zhang et al., 2018), the peak in photosynthetic activity (Liu et al., 2023c), and the stabilization of ecosystem respiration processes, which encompass both plant and soil respiration (Liu et al., 2023b). Numerous studies have shown that meteorological factors are the dominant drivers of these phenomena in alpine regions (Chen et al., 2020b; Wang et al., 2020). As shown in **Figure 5**, the peak in productivity and carbon sink capacity at both the vegetation and ecosystem levels had the shortest lag with temperature (less than one month), followed by precipitation, and had the longest lag with solar radiation (approximately three months). This is generally consistent with the findings of Hossain et al. (2021) and Li et al. (2023) in alpine grassland regions.

In past studies on the lag effects of meteorological factors on ecosystem vegetation productivity (or carbon sink capacity) primarily employed simple correlation analysis methods (e.g. Pearson correlation analysis or linear correlation analysis.) (Lyu et al., 2023; Tian et al., 2023; Huang et al., 2024; Jia et al., 2024), and without accounting for the multi-collinearity between different meteorological factors and the control of confounding variables. To eliminate the complex interactions and dependencies among independent variables and to reveal the true relationships between NPP (or NEP) and meteorological factors, this study employed partial correlation coefficients. This method was used to reflect the driving relationship of meteorological factors on NPP (or NEP). After applying the time lag treatment, the absolute PCC values between meteorological factors and NPP (or NEP) increased to varying degrees. This is primarily related to the differences in sensitivity to meteorological factors among different PFTs (Liu et al., 2015). After applying the time lag treatment to the meteorological factors, the variability of PCC values among different PFTs increased, particularly when analyzing the partial correlation between carbon sink capacity and temperature, as well as between carbon sink capacity and solar radiation from an ecosystem perspective. The ranking of PCC values for different PFTs indicates their correlation with various meteorological factors and their sensitivity to changes (**Table 2**) (Liu et al., 2015). Therefore, when analyzing solely from the vegetation level, the differences in the sensitivity to changes in meteorological factors (Tem, Pre, and SR) for the vegetation productivity (NPP) of different PFTs are not significant. However, from an ecosystem perspective, the sensitivity of ecosystem carbon sink capacity (NEP) to meteorological factors (Tem and SR) under different PFTs are ranked as follows: SVL > C3A > PW > BDS > C3. This indicates that the differences in sensitivity are not due to the vegetation itself, but rather to the variations in the ecosystem environments dominated by different PFTs. This is related to differences in ecosystem structure and function among different PFTs. For instance, ecosystems with deeper root systems and higher soil organic matter content tend to exhibit greater resistance to short-term climate variations (Jackson et al., 1996; Jobbágy and Jackson, 2000), which explained why sparsely vegetated lands (SVL) are the most sensitive to changes in meteorological factors.

### 4.3 Spatial variations in time-lag effects

The lag between Tem and NPP is primarily related to soil temperature and nutrient availability (Braswell et al., 1997; Kong et al., 2020). In addition to these factors, the lag between Tem and NEP also involved soil microbial respiration and organic matter decomposition (Braswell et al., 1997; Liu et al., 2022). The study results indicate that the lag effects of both NPP and NEP with temperature are significantly negatively correlated with longitude and significantly positively correlated with elevation (**Table 3**). Most areas of the northwestern SCR, characterized by higher elevations, vegetation growth exhibits a lag of more than 20 days relative to temperature changes, while the carbon sink response of the ecosystem lags by more than 15 days. In contrast, in the lower elevation eastern SYR region, both vegetation growth and the ecosystem’s carbon sink lag behind temperature changes by less than 10 days. This is closely related to the regional characteristics of the study area (with relatively high elevations and lower temperatures in the western SCRYR region). The slower warming rate in these areas means that it takes longer for plants to transition from dormancy to active growth (Zheng et al., 2021; Rathore et al., 2022). Plants in high-elevation regions are typically adapted to cold environments, exhibiting traits such as slower growth rates, shorter growing seasons, and lower metabolic rates (Gale, 2004; Kumar and Vats, 2017; Kumar et al., 2023). Additionally, in the high- elevation regions of the western part of the study area, the lag time of NEP relative to NPP in response to temperature is shorter. This suggests that ecosystem productivity is more sensitive to temperature variations than vegetation productivity. For instance, processes such as soil microbial activity and associated biochemical reactions exhibit a more rapid response to temperature changes compared to vegetation (Zifcakova, 2020; Wang et al., 2021a).

The lag effect between NPP and Pre indicated the temporal delay between precipitation events and the subsequent availability of water to plant roots, representing the time required for water to percolate and reach the root zone (Jobbágy et al., 2002; Kong et al., 2020). Furthermore, the lag effect between NEP and Pre encompasses the additional delay associated with soil microbial responses, such as respiration and decomposition, to the infiltration of precipitation into the soil (Zhang et al., 2013; Wang et al., 2023a). On a temporal scale, although the lag range of NPP with Pre (**Figure 9E**, 0 to 60 days) was longer compared to the lag range of NEP with Pre (**Figure 10E**, 0 to 48 days), the spatial extent of the long lag effect between NEP and Pre was more widespread. Specifically, the lag time of NEP with Pre exceeded 30 days in 80% of the study area (**Figure 10B**), encompassing nearly the entire SCR region and much of the SYR region. In contrast, the lag time of NPP with Pre exceeding 30 days only covers 25% of the area, mainly concentrated in the northwestern part of the SCR region (**Figure 9B**). The reasons for this phenomenon are twofold. First, there is a precipitation gradient across the study area (**Supplementary Figure S7B**), increasing from the northwest to the southeast. Second, the northwestern edge of the SCR region is characterized by higher elevations, lower vegetation density (with a higher proportion of SVL), poor soil quality, and slower infiltration rates (Chen et al., 2020a). These factors make it more challenging for plant roots to access water (Kong et al., 2020), thereby contributing to the concentration of the long lag effect between NPP and Pre in this region. The broader distribution of the long lag effect between NEP and Pre, as observed, aligns closely with regions above 4500 meters in elevation (as indicated by the correlation coefficients in **Table 3**). This suggests that, in comparison to simpler vegetation systems, the complex ecosystems at high elevations are less responsive to changes in Pre concerning carbon sequestration (or carbon emissions). This is related to the limitations that low temperatures in high- elevation areas impose on microbial activity (Wang et al., 2024). Even with changes in precipitation, the response of soil microbial activity is relatively slow, resulting in a lower sensitivity of ecosystem carbon sequestration to precipitation changes (Zeng et al., 2022; Wang et al., 2024).

The lag effect between NPP and SR indicated a delayed response of plants to changes in solar radiation, affecting photosynthetic efficiency and carbon fixation processes (Li et al., 2023; Jia et al., 2024). This includes the adjustment of photosynthesis, biomass accumulation processes, seasonal variations, and non-light-limiting factors within the ecosystem (Buermann et al., 2018; Li et al., 2023). Compared to other meteorological factors, the lag effect of NPP in response to SR is the longest, with lag times reaching around 60 days within the study area, which is consistent with results from other studies conducted in alpine grassland regions (Li et al., 2023). Furthermore, this long lag effect was minimally influenced by spatial factors (**Table 3**) and PFTs (**Table 4**). This suggests that precipitation and temperature are the primary meteorological factors limiting NPP, rather than solar radiation. Even if solar radiation increases, improvements in NPP may be limited if other factors do not improve, especially in areas with low temperatures, arid conditions, or poor soil quality (Knapp et al., 2014). The study found that the lag effect between NEP and SR exhibited a bimodal pattern. Areas with short lag effects (less than 10 days) closely overlap with carbon source regions (NEP <0), while areas with long lag effects (over 55 days) closely overlap with carbon sink regions (NEP > 0). In carbon source regions with low vegetation cover, low biomass, and high rates of organic matter decomposition in the soil, ecosystems are typically in a state of carbon emission (Wu et al., 2022; Zeng et al., 2023). Soil respiration and organic matter decomposition respond quickly to environmental changes, and microbial activity can react to changes in photosynthetic energy within a short time frame (Wu et al., 2022; Ma et al., 2024; Zhao et al., 2024). Carbon sink regions typically have higher vegetation cover and biomass (Wu et al., 2022; Zhao et al., 2024) result in a time-lag effect between NEP and SR that mainly depends on the lag time of vegetation response to SR (i.e., the long time-lag effect between NPP and SR).

### 4.4 PFTs variations in time-lag effects

Different PFTs exhibit substantial variations in the lag effects of NPP and NEP in response to meteorological factors due to differences in their physiological characteristics, as well as associated variations in soil types and microbial communities (Weng et al., 2021; Koranda et al., 2023). Aside from the lag effect between NPP and SR, which did not show significant differences among different PFTs, the lag effects of NPP (or NEP) in response to other meteorological factors exhibited noticeable variations across different PFTs (**Table 4**). The primary reasons for this outcome are twofold. Firstly, the sensitivity of NPP (or NEP) to changes in meteorological factors varies across different PFTs (as detailed in Section 4.1). On the other hand, we found that this is also closely related to the spatial distribution of vegetation. The more concentrated the vegetation distribution within the study area, the more similar the lag effects of NPP (or NEP) with meteorological factors. Conversely, the more dispersed the vegetation distribution, the greater the differences in lag effects. For example, the lag time of NPP for C3A with Tem is within 10 days in the SYR region, while it exceeds 20 days in the SCR region. Similar patterns are observed for other PFTs with different meteorological factors. This indicated that different PFTs and their respective environments exhibited corresponding adaptations to changes in meteorological factors (Wang and Ni, 2005; Rao et al., 2024). The consistent impact of regional microclimatic conditions (especially temperature and precipitation) on vegetation within the area is greater than the differences in physiological characteristics of the plants themselves (Jones, 1993; Pincebourde et al., 2016).

## 5 Conclusions

This study highlighted the significant spatiotemporal dynamics of NPP and NEP within the SCRYR region, both at the vegetation level and the broader ecosystem level. It revealed complex interactions with meteorological factors such as temperature, precipitation, and solar radiation. The findings underscore the distinct responses of different PFTs to these factors, influenced by both physiological characteristics and environmental conditions. Our analysis demonstrates that, while solar radiation exerts the longest lag effect on NPP, temperature and precipitation are the primary drivers of carbon sink capacity, as evidenced by the pronounced sensitivity of NEP and NPP to these variables. The observed lag effects, particularly the bimodal patterns between NEP and SR, emphasize the differential carbon dynamics across regions, with shorter lags corresponding to carbon source areas and longer lags to carbon sink areas.

The study also identified that ecosystem responses to climatic changes are more heavily influenced by regional microclimatic conditions than by the physiological traits of the vegetation alone. This is particularly evident in high- elevation areas where the slower warming rates and unique environmental conditions lead to distinct lag responses. The implications of these findings are critical for understanding the carbon dynamics in alpine ecosystems and can inform future conservation and management strategies aimed at mitigating climate change impacts. Overall, our results suggest that effective ecosystem management must consider both the immediate and lagged responses of different PFTs to changing meteorological conditions, particularly in regions with varied microclimatic and environmental contexts.

## Data Availability

The original contributions presented in the study are included in the article/**Supplementary Material**. Further inquiries can be directed to the corresponding author.
