# Supplementary material for: Time-lag effects of NEP and NPP to meteorological factors in the source regions of the Yangtze and Yellow Rivers
Source: Front Plant Sci. 2025 Jan 10;15:1502384. doi: 10.3389/fpls.2024.1502384 (PMC11757257; doi:10.3389/fpls.2024.1502384)
Supplement: Supplementary file 1 [file Table1.docx]

Supplementary Material

# Supplementary Figures


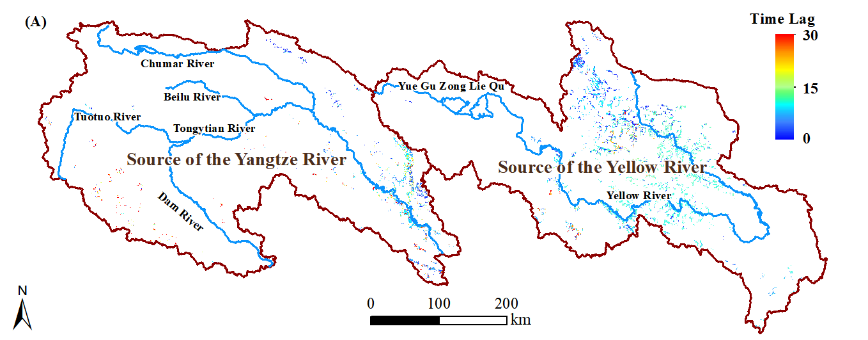

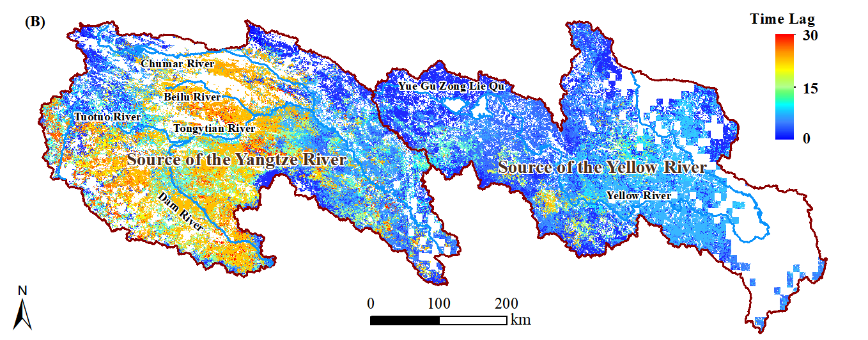

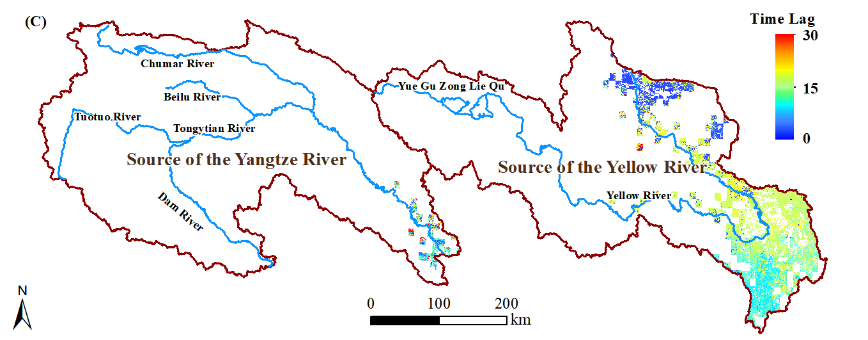

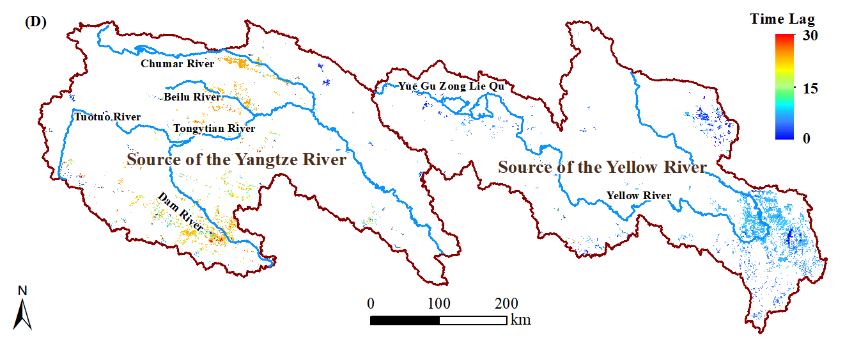

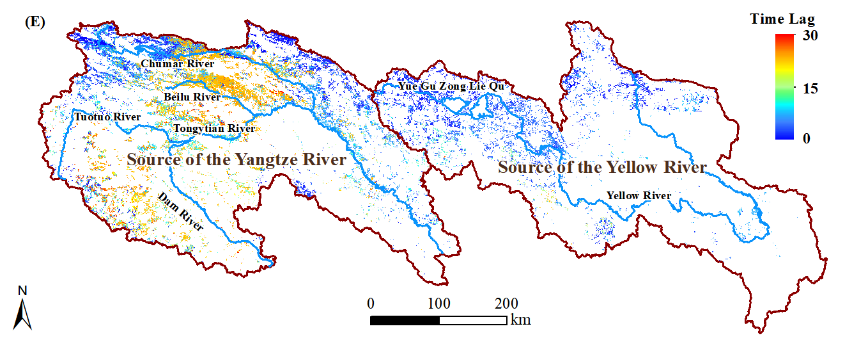

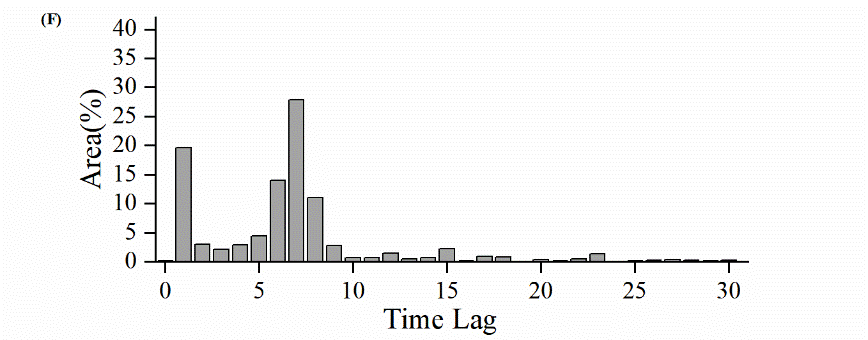

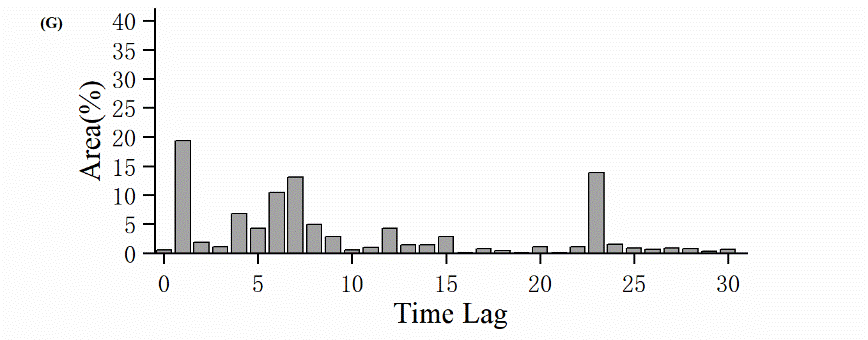

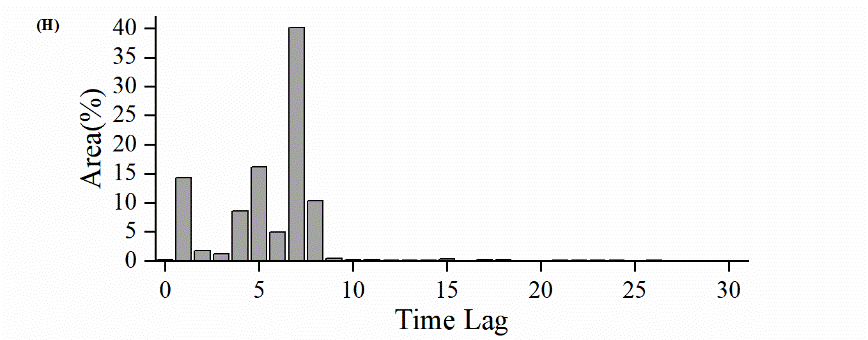

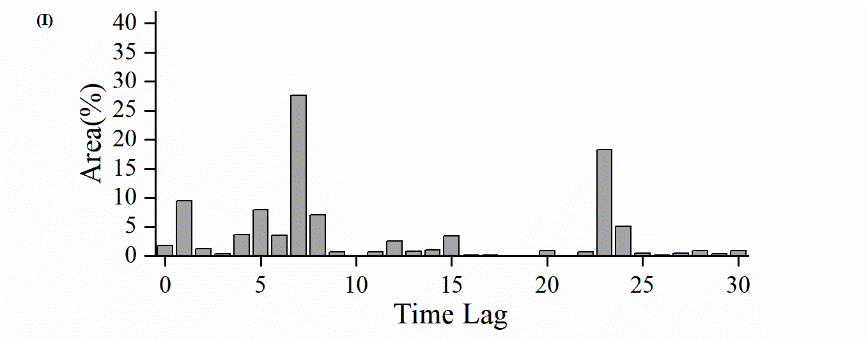

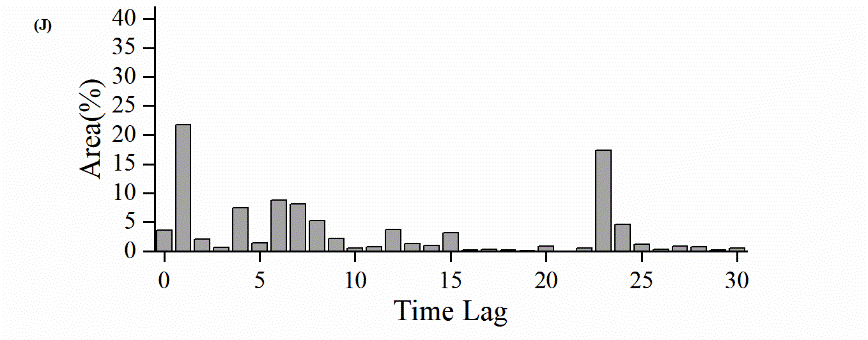


**Fig. S1** Time lags at which there was maximum PCC between the NPP and Tem for different PFTs.

Note: (A) and (F) represents the spatial distribution and histogram of time lags for BDS, respectively; (B) and (G) represents the spatial distribution and histogram of time lags for C3A, respectively; (C) and (H) represents the spatial distribution and histogram of time lags for C3, respectively; (D) and (I) represents the spatial distribution and histogram of time lags for PW, respectively; (E) and (J) represents the spatial distribution and histogram of time lags for SVL, respectively.


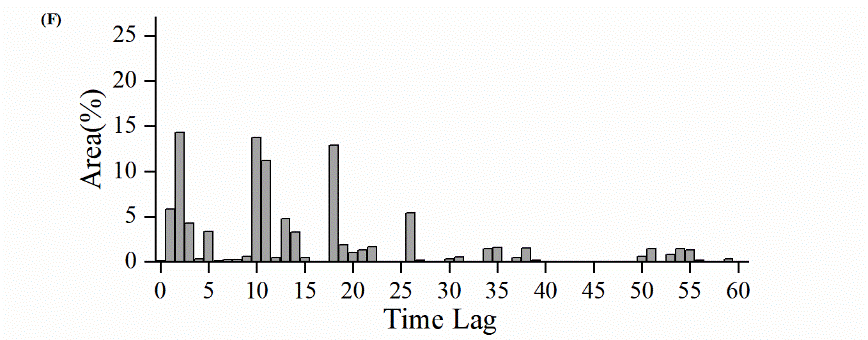

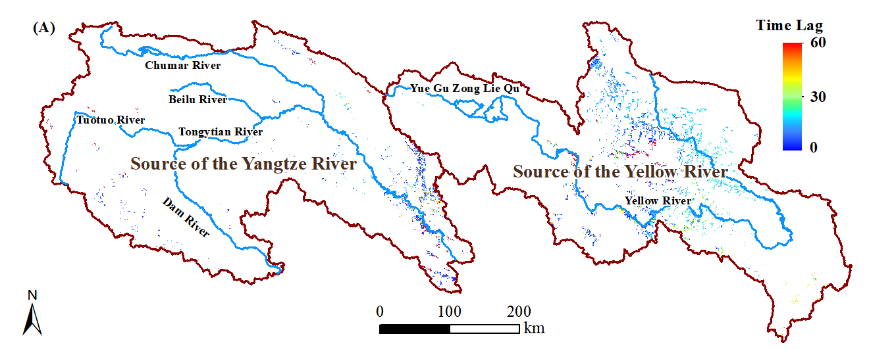

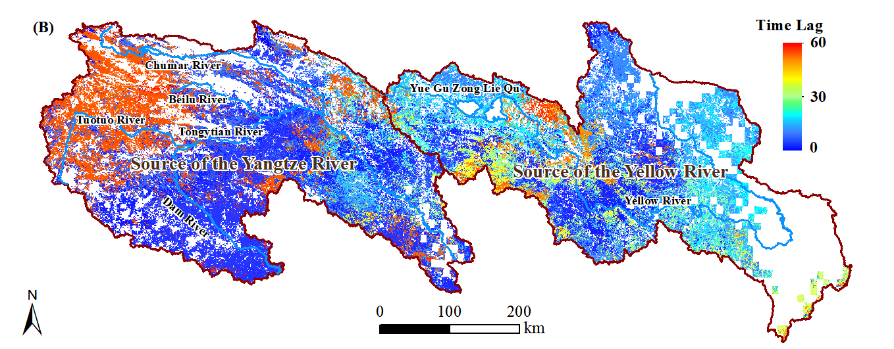

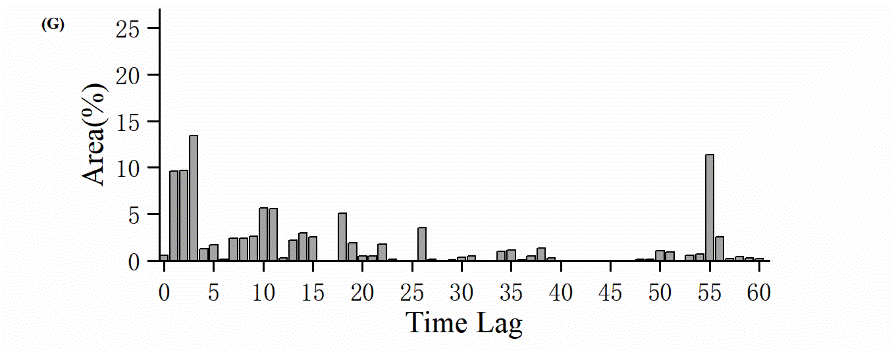

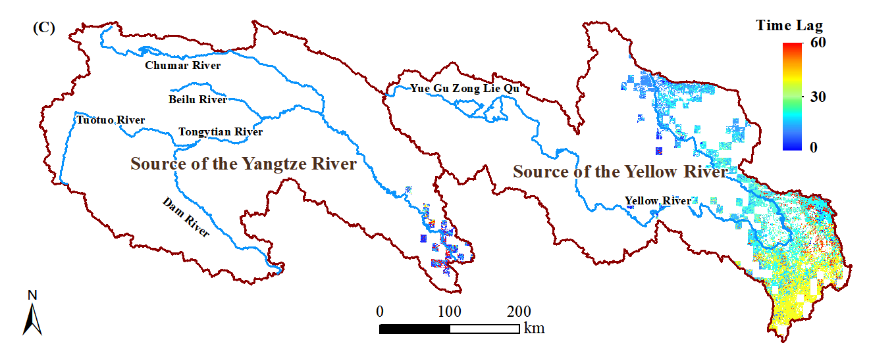

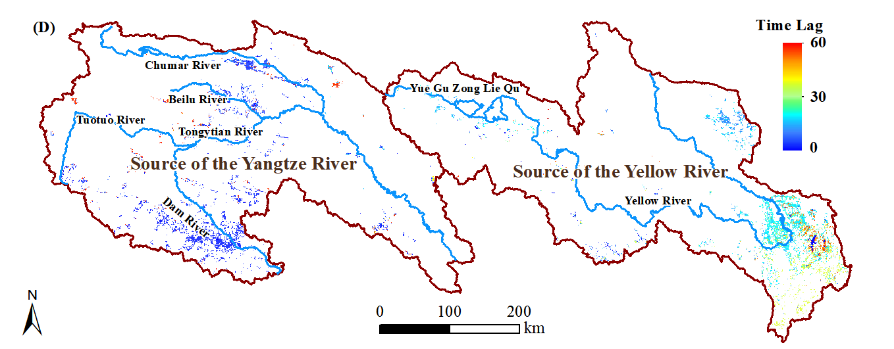

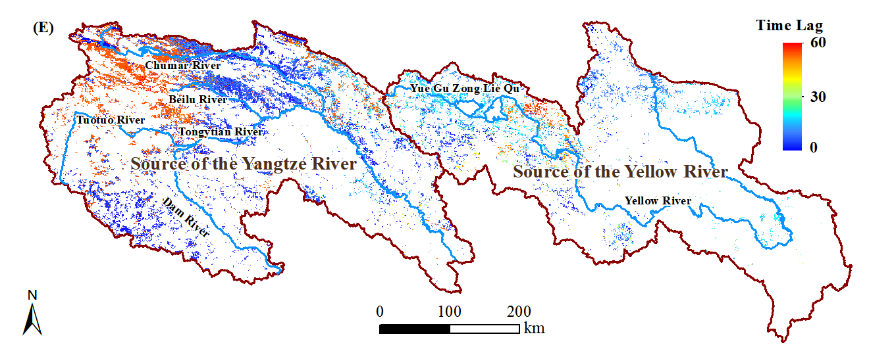

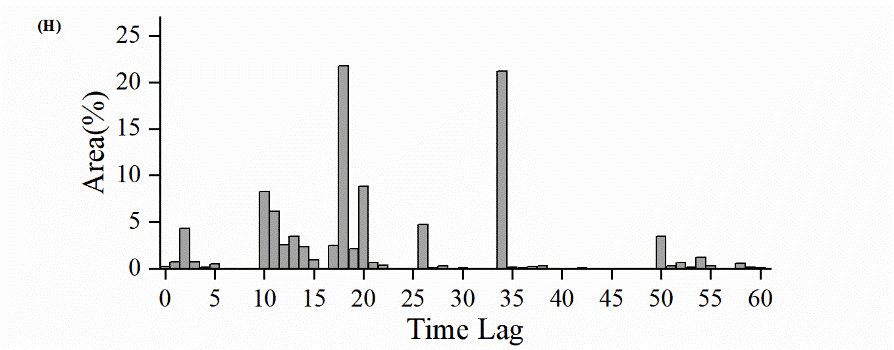

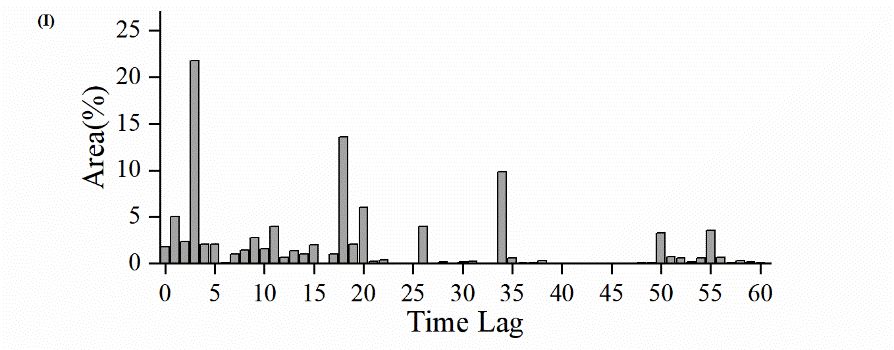

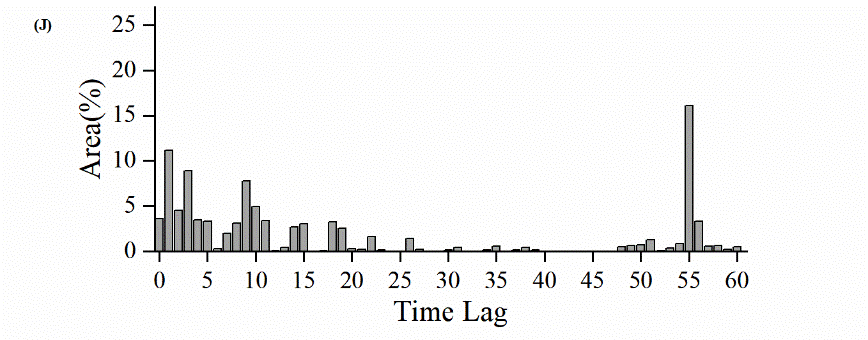


**Fig. S2** Time lags at which there was maximum PCC between the NPP and Pre for different PFTs.

Note: (A) and (F) represents the spatial distribution and histogram of time lags for BDS, respectively; (B) and (G) represents the spatial distribution and histogram of time lags for C3A, respectively; (C) and (H) represents the spatial distribution and histogram of time lags for C3, respectively; (D) and (I) represents the spatial distribution and histogram of time lags for PW, respectively; (E) and (J) represents the spatial distribution and histogram of time lags for SVL, respectively.


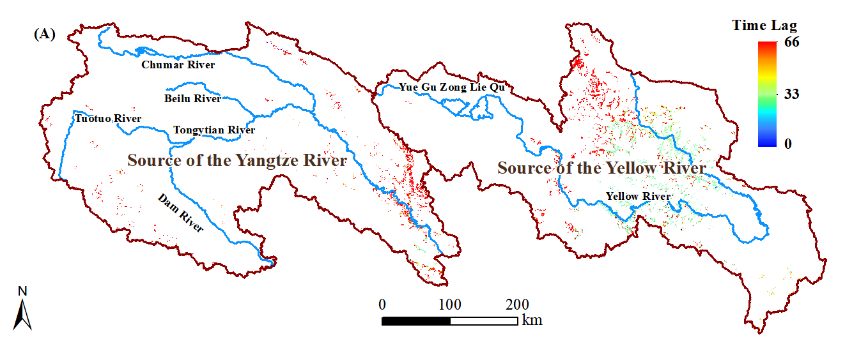

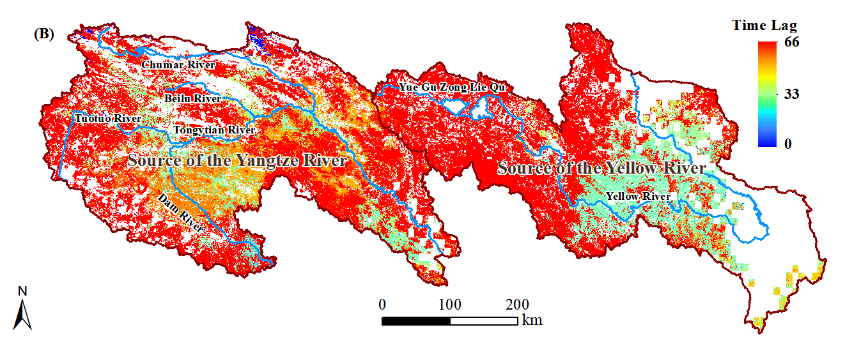

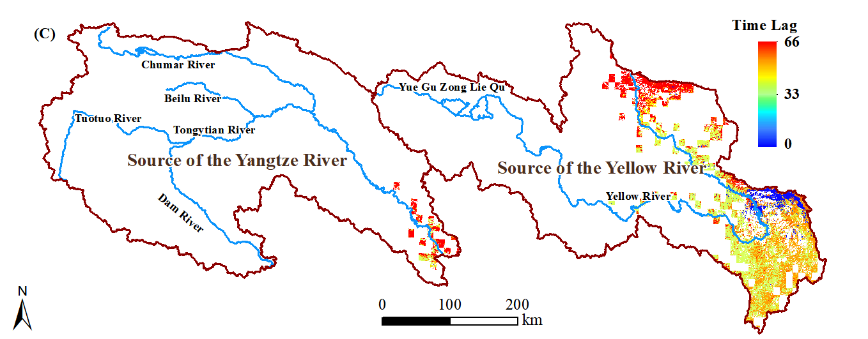

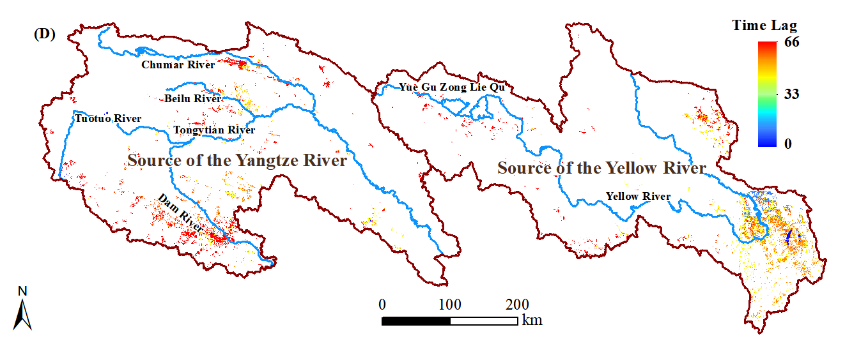

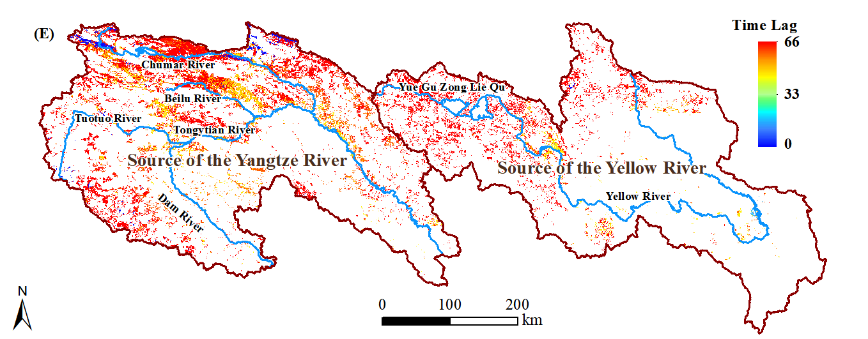

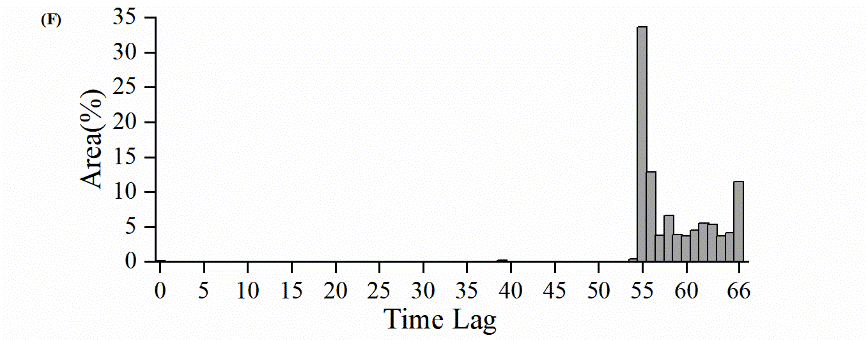

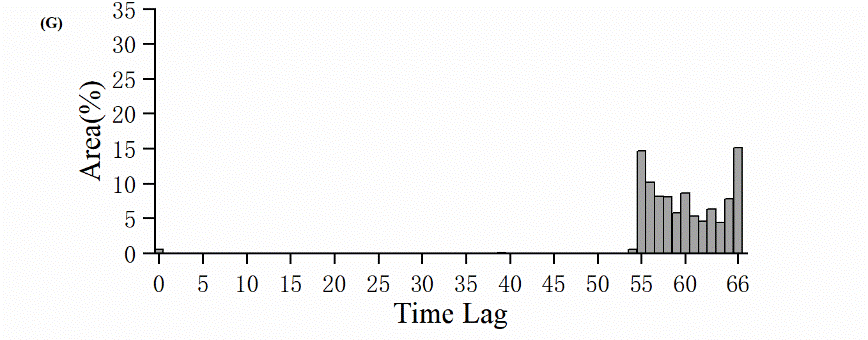

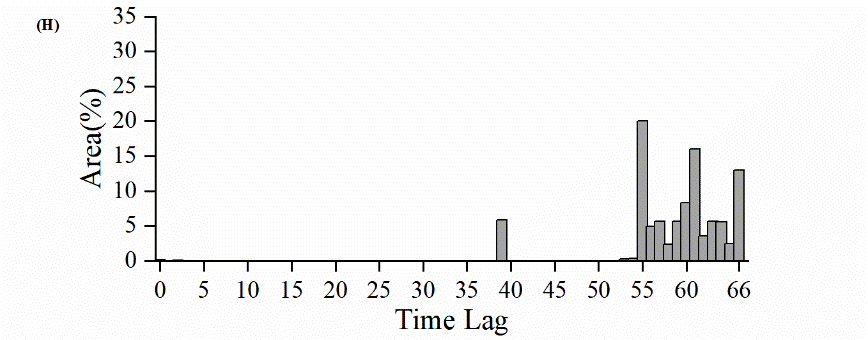

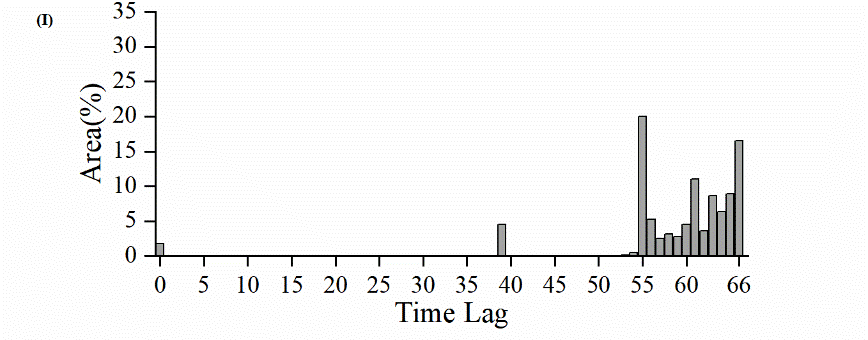

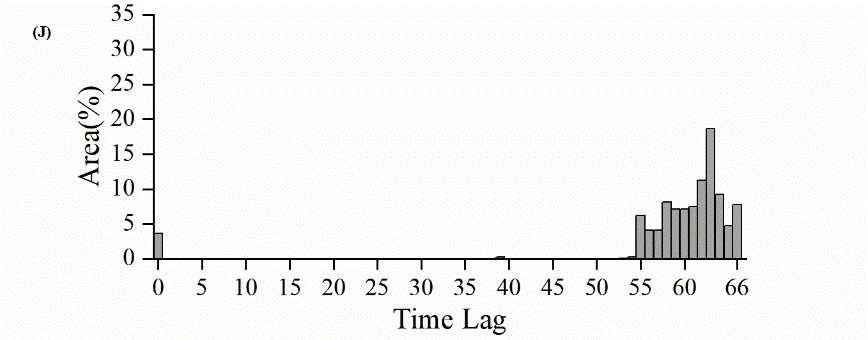


**Fig. S3** Time lags at which there was minimum PCC between the NPP and SR for different PFTs.

Note: (A) and (F) represents the spatial distribution and histogram of time lags for BDS, respectively; (B) and (G) represents the spatial distribution and histogram of time lags for C3A, respectively; (C) and (H) represents the spatial distribution and histogram of time lags for C3, respectively; (D) and (I) represents the spatial distribution and histogram of time lags for PW, respectively; (E) and (J) represents the spatial distribution and histogram of time lags for SVL, respectively.


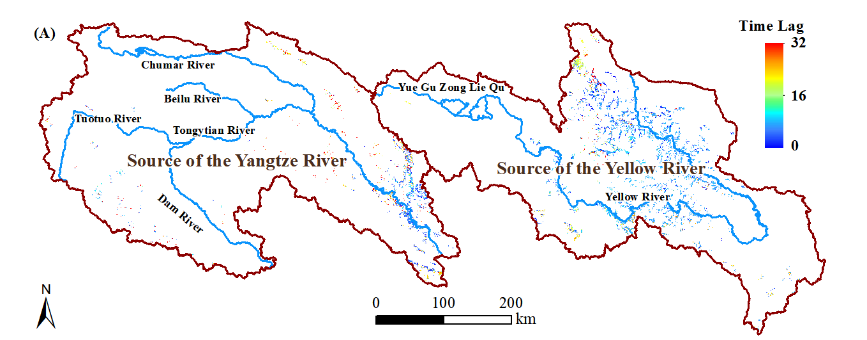

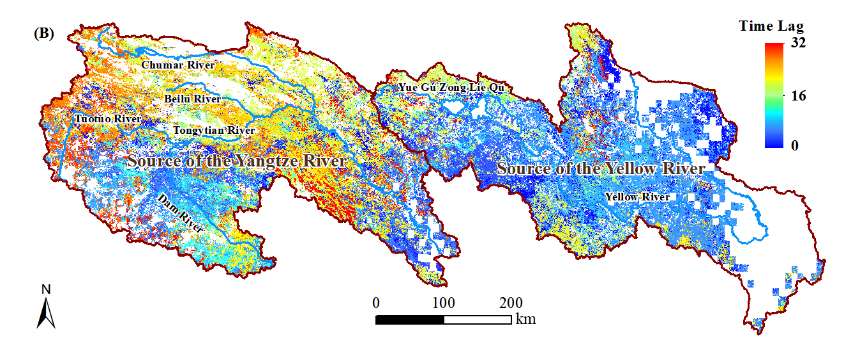

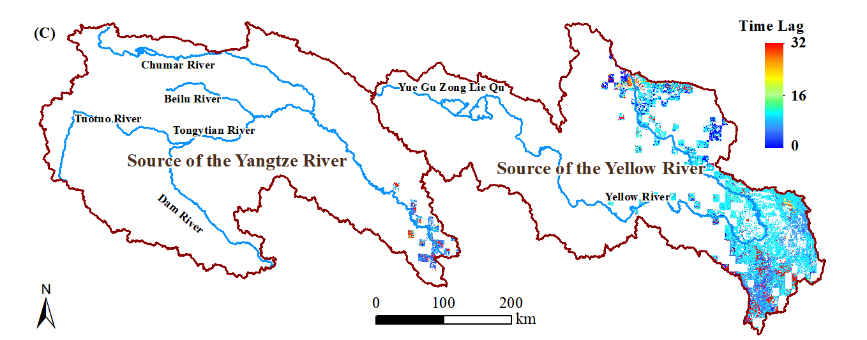

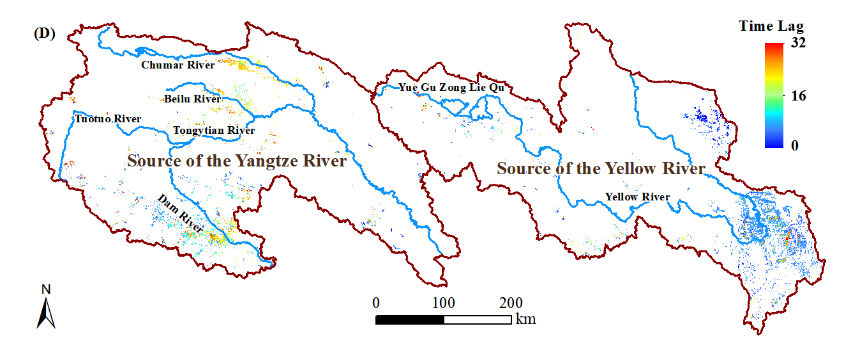

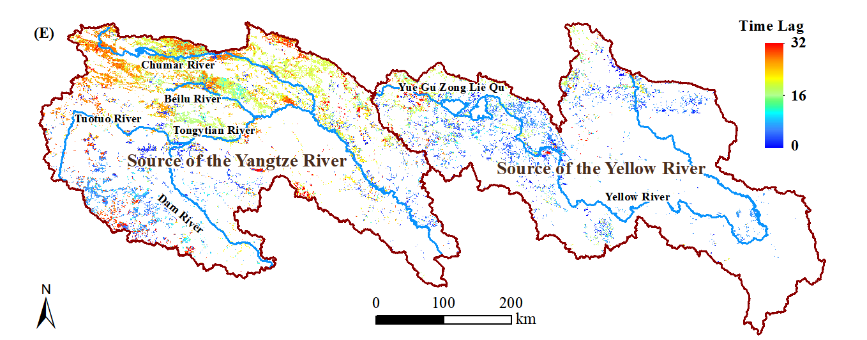

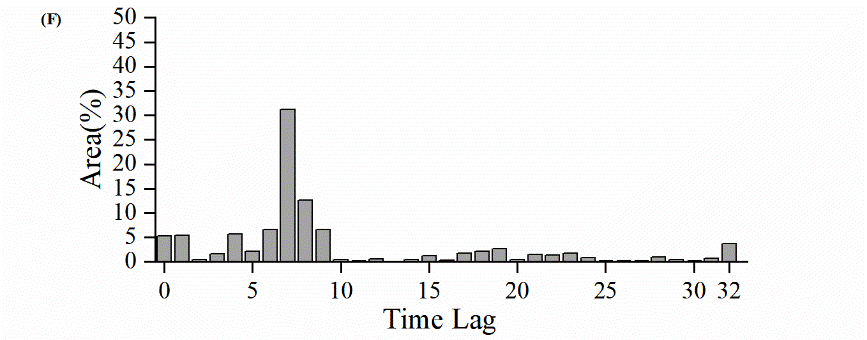

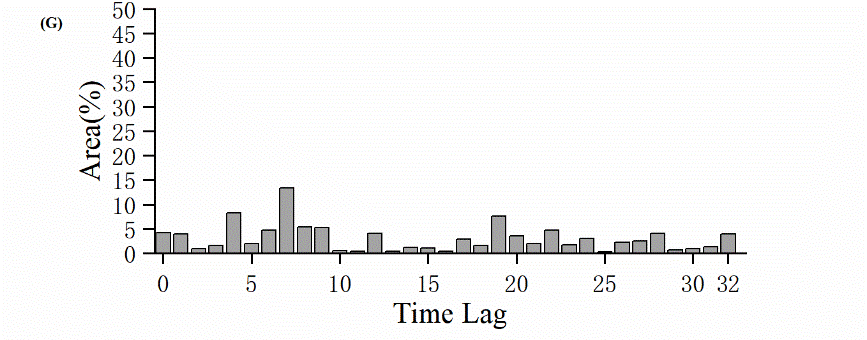

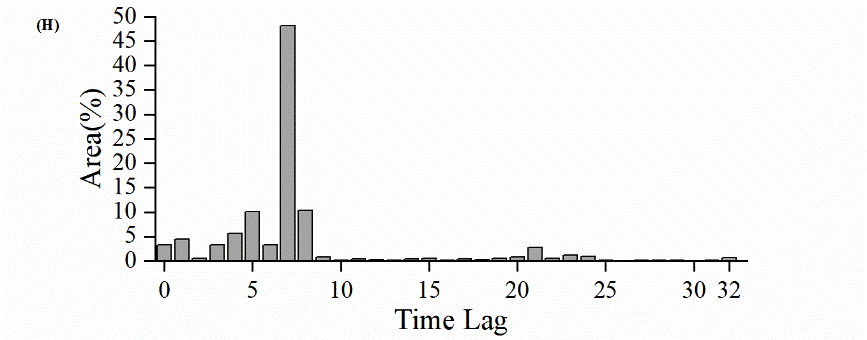

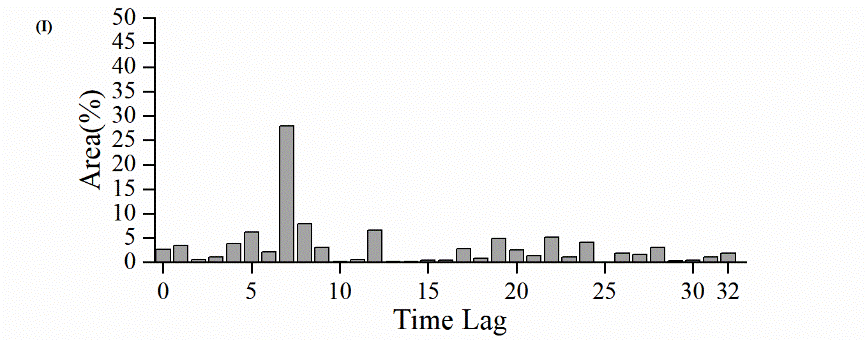

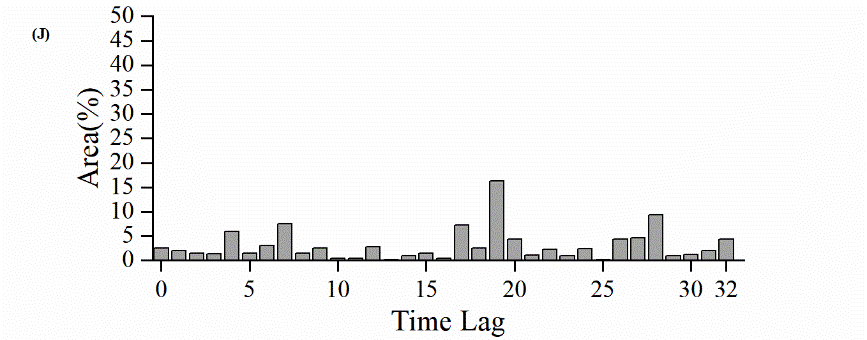


**Fig. S4** Time lags at which there was maximum PCC between the NEP and Tem for different PFTs.

Note: (A) and (F) represents the spatial distribution and histogram of time lags for BDS, respectively; (B) and (G) represents the spatial distribution and histogram of time lags for C3A, respectively; (C) and (H) represents the spatial distribution and histogram of time lags for C3, respectively; (D) and (I) represents the spatial distribution and histogram of time lags for PW, respectively; (E) and (J) represents the spatial distribution and histogram of time lags for SVL, respectively.


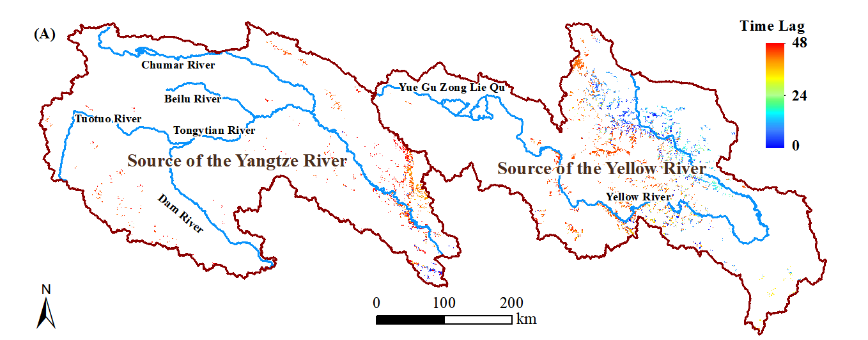

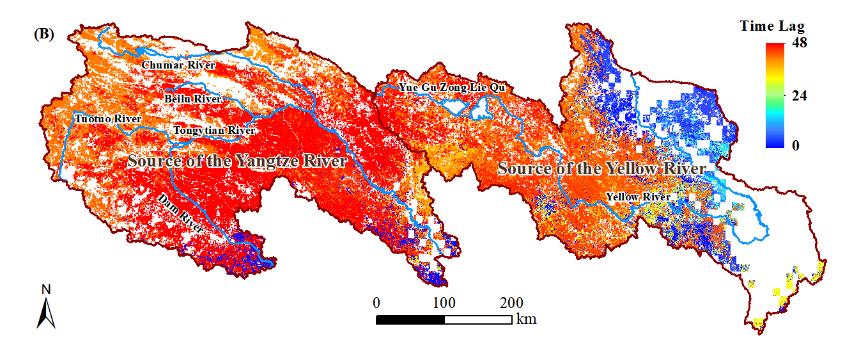

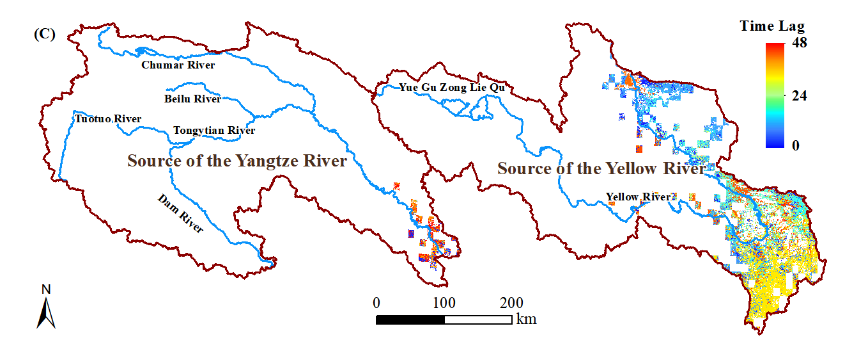

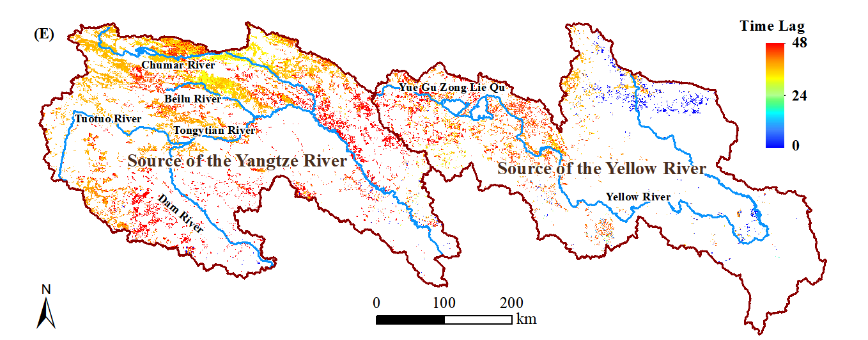

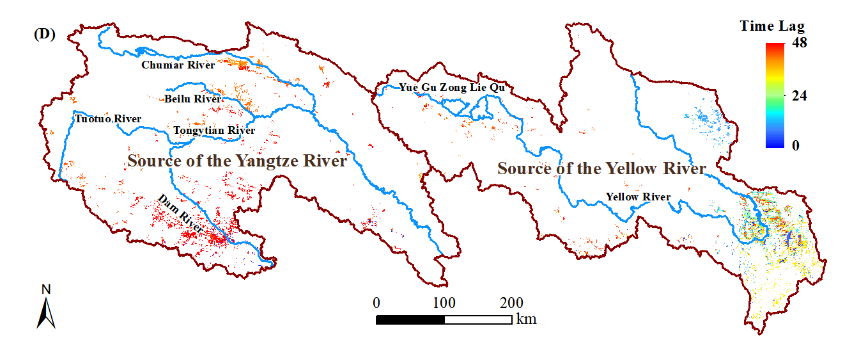

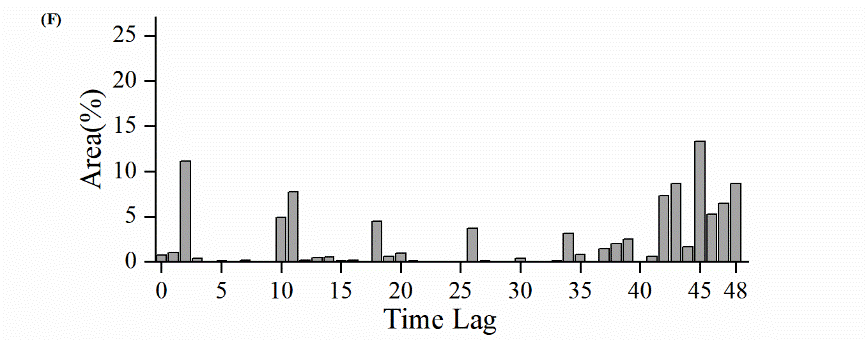

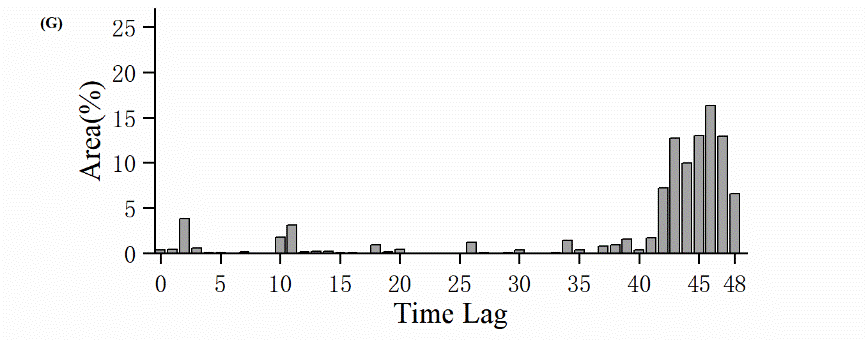

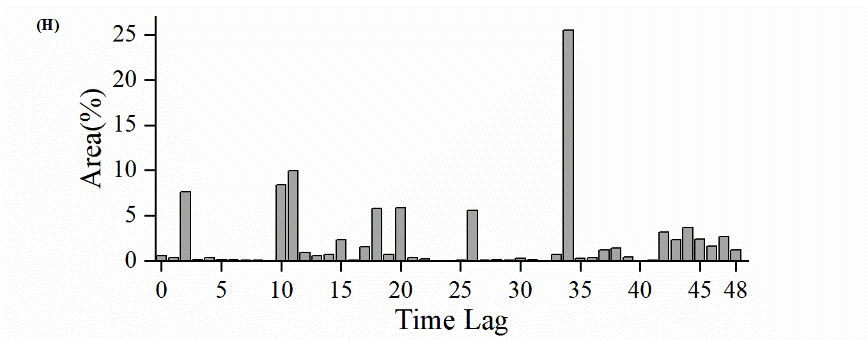

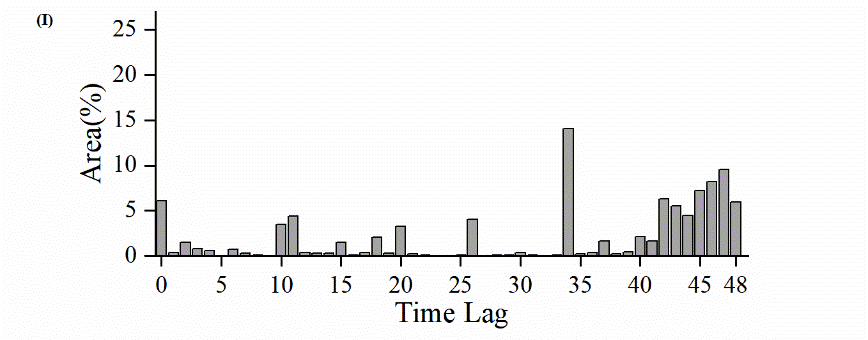

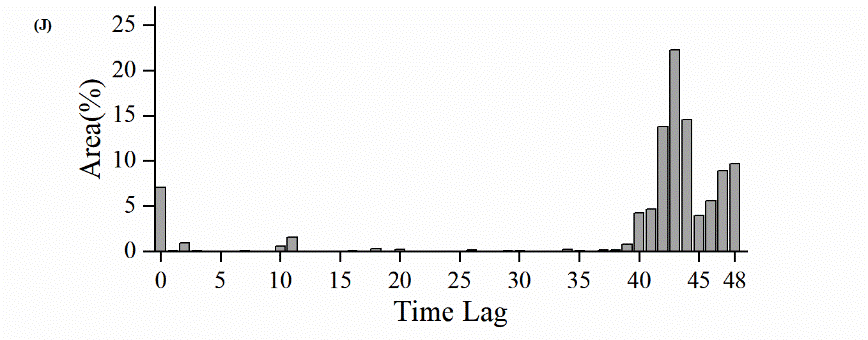


**Fig. S5** Time lags at which there was maximum PCC between the NEP and Pre for different PFTs.

Note: (A) and (F) represents the spatial distribution and histogram of time lags for BDS, respectively; (B) and (G) represents the spatial distribution and histogram of time lags for C3A, respectively; (C) and (H) represents the spatial distribution and histogram of time lags for C3, respectively; (D) and (I) represents the spatial distribution and histogram of time lags for PW, respectively; (E) and (J) represents the spatial distribution and histogram of time lags for SVL, respectively.


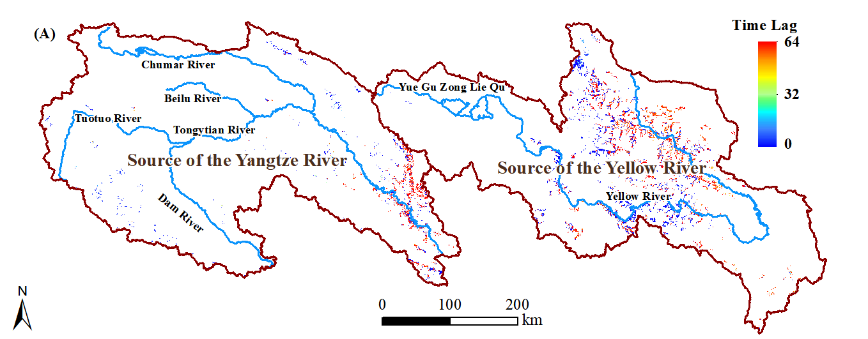

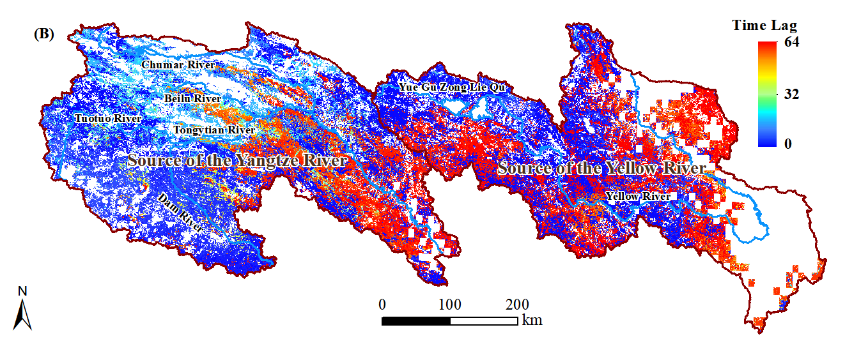

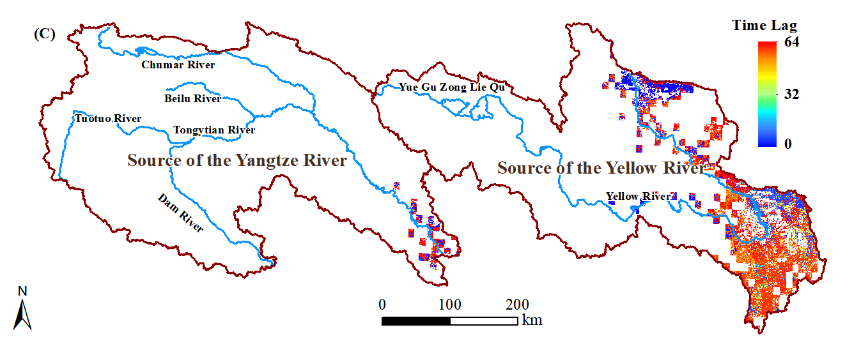

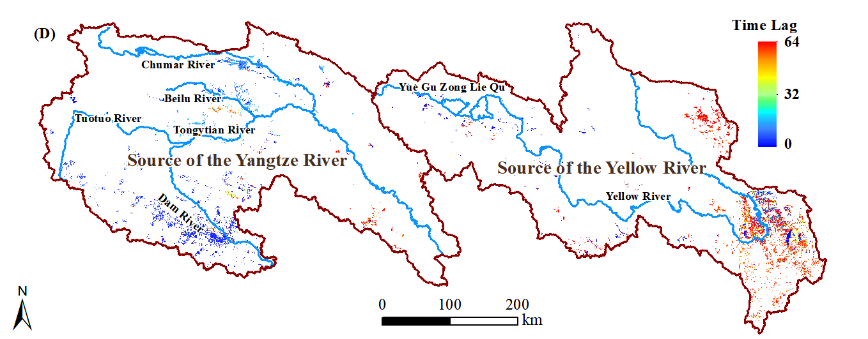

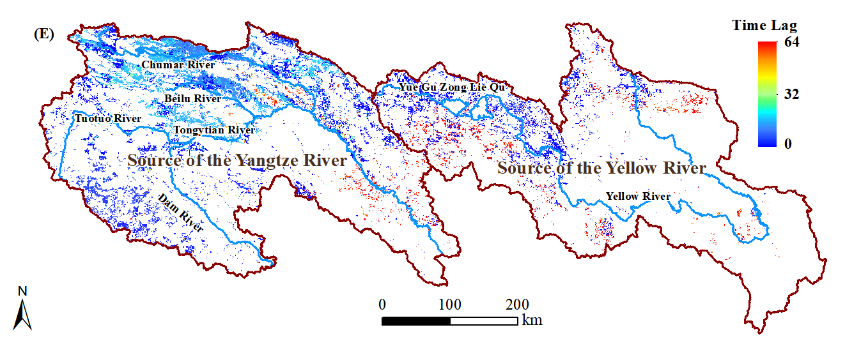

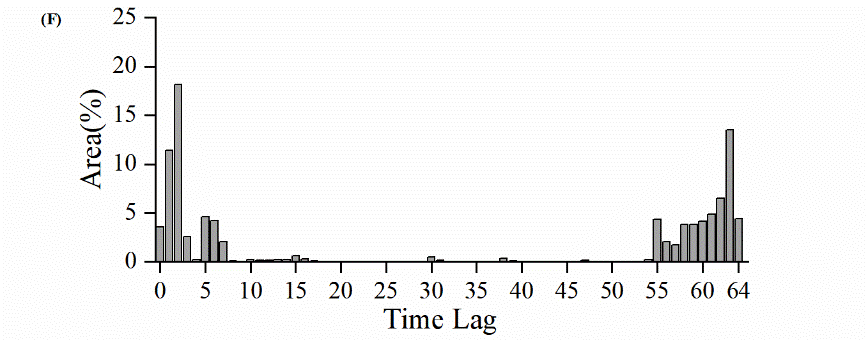

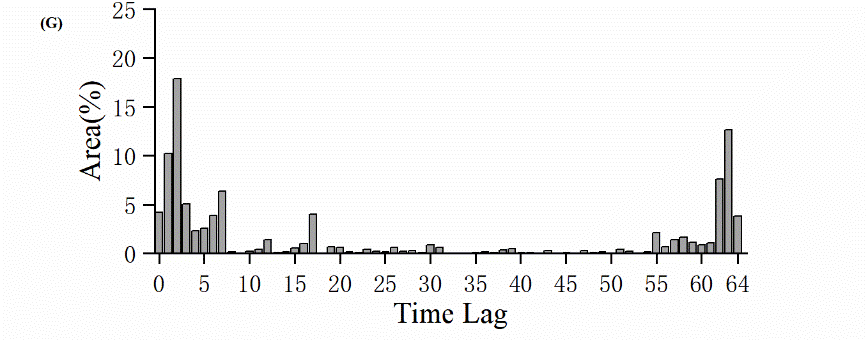

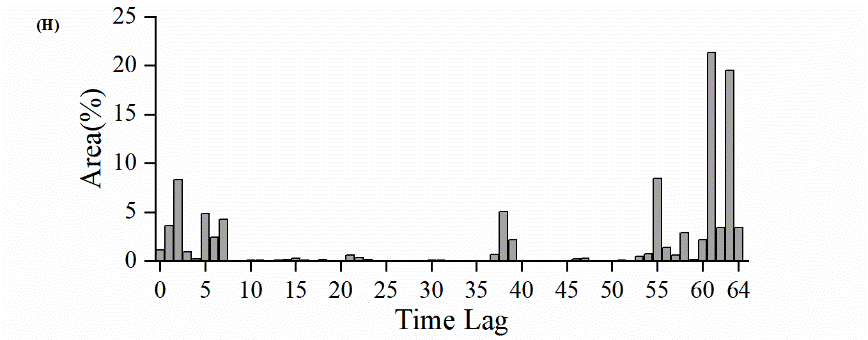

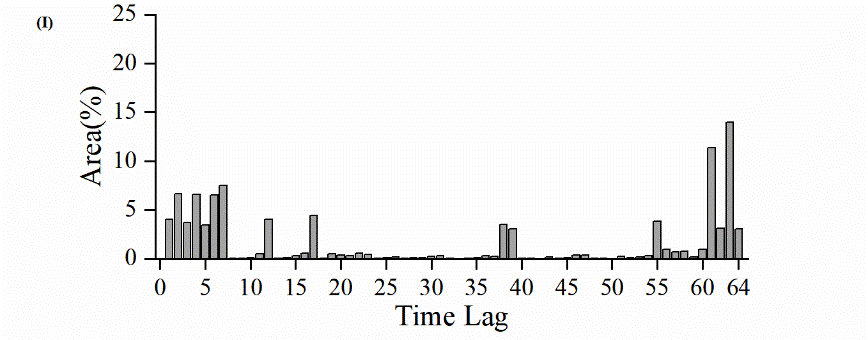

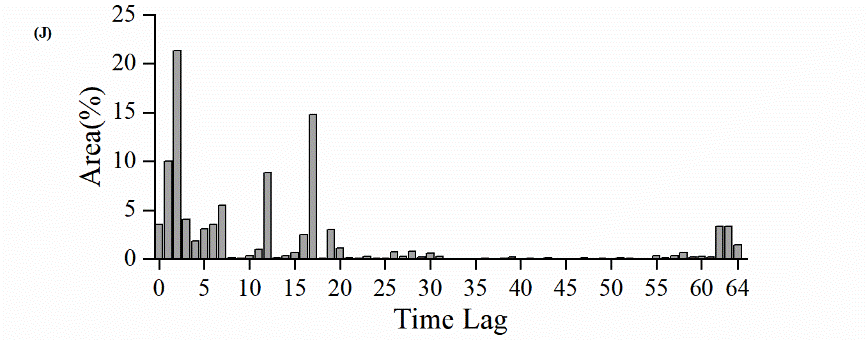


**Fig. S6** Time lags at which there was minimum PCC between the NEP and SR for different PFTs.

Note: (A) and (F) represents the spatial distribution and histogram of time lags for BDS, respectively; (B) and (G) represents the spatial distribution and histogram of time lags for C3A, respectively; (C) and (H) represents the spatial distribution and histogram of time lags for C3, respectively; (D) and (I) represents the spatial distribution and histogram of time lags for PW, respectively; (E) and (J) represents the spatial distribution and histogram of time lags for SVL, respectively.


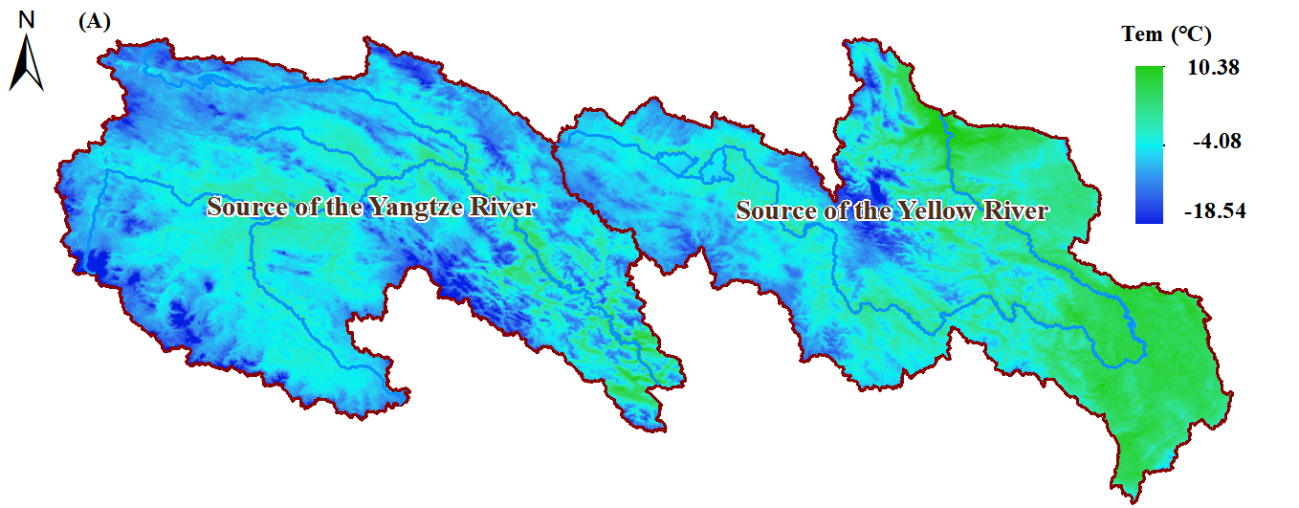

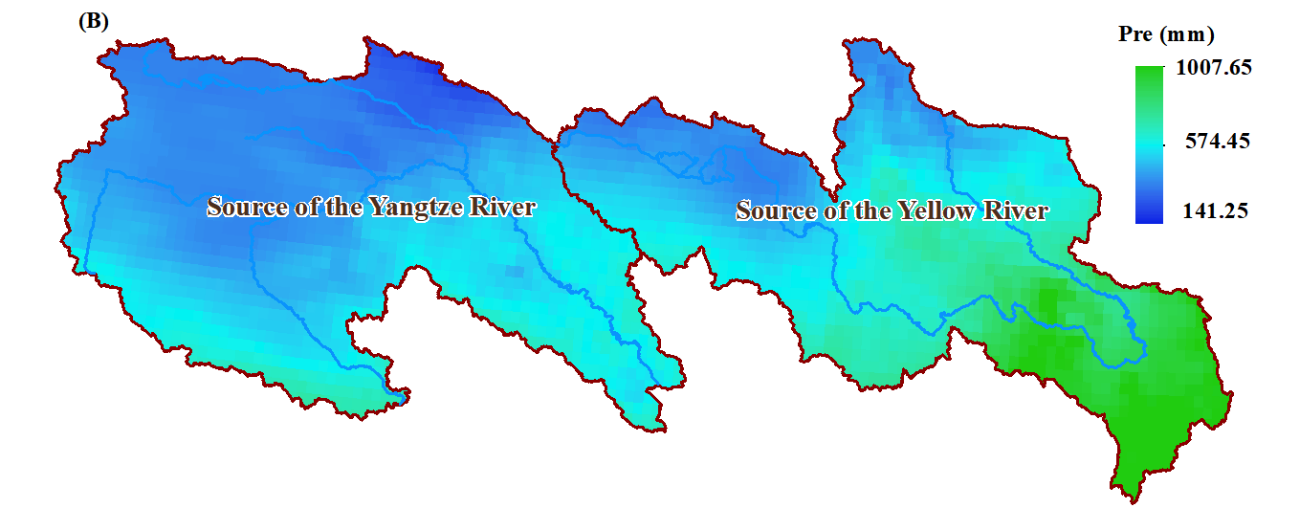

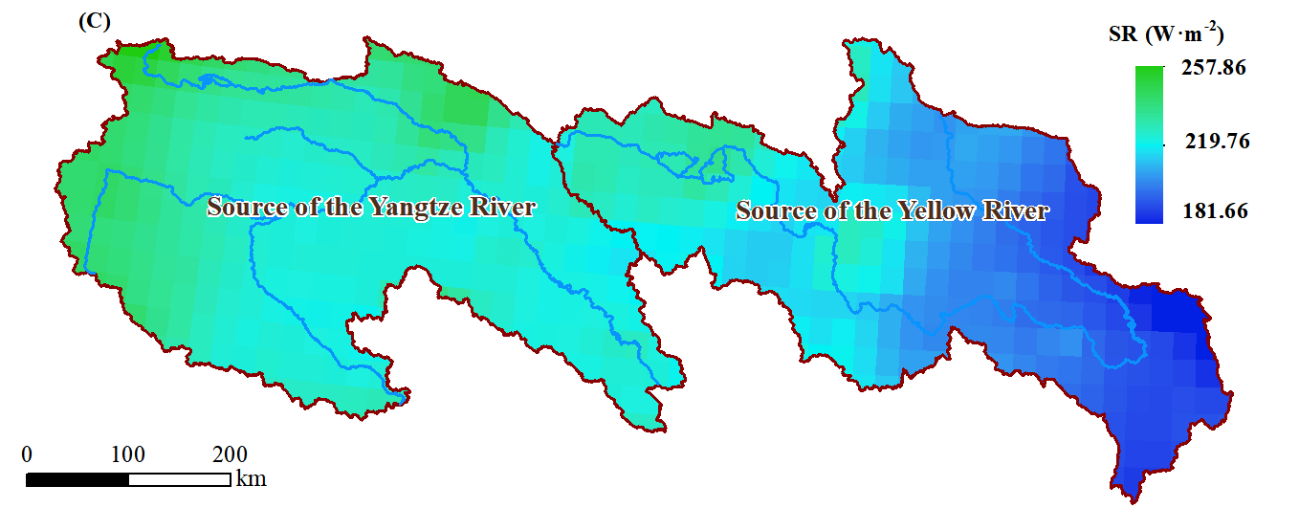


**Fig. S7** The annual Tem (℃), Pre (mm), and SR (W·m^-2^) from 2015 to 2020
